# B Cell Subsets and Atherosclerosis: Updates and Emerging Concepts

**DOI:** 10.1111/imr.70124

**Published:** 2026-04-24

**Authors:** Sophieke Lems, Megan Grace Mazzei, Amanda C. Foks, Coleen A. McNamara

**Affiliations:** ^1^ Division of BioTherapeutics, Leiden Academic Centre for Drug Research Leiden University Leiden the Netherlands; ^2^ Beirne B. Carter Center for Immunology Research University of Virginia Charlottesville Virginia USA; ^3^ Division of Cardiovascular Medicine, Department of Medicine University of Virginia Charlottesville Virginia USA

**Keywords:** atherosclerosis, B cells, immunoglobulins, immunotherapy, oxidation‐specific epitopes

## Abstract

Atherosclerotic cardiovascular disease (ASCVD) is increasingly recognized not just as a lipid‐driven disease, but as a complex interplay between vascular cells and the immune system. Accumulating evidence highlights a central, yet heterogeneous role for B cells in atherogenesis, with distinct subsets displaying opposing roles. In this review, we provide an in‐depth overview of the contributions of B cell subsets to ASCVD, including emerging insights into the roles and pathways of atheroprotective innate B cells producing IgM against oxidation‐specific epitopes (IgM^OSE^) and newly appreciated age‐associated B cells (ABCs), a distinct subset that accumulates with aging and potentially exacerbates atherosclerosis. By integrating insights from preclinical models and human studies, we describe the mechanisms through which B cell subsets influence ASCVD, including antigen presentation and immune checkpoint‐mediated communication, secretion of cytokines and chemokines, and we highlight that humoral immunity in atherosclerosis reflects a context‐dependent interplay between antibody effector properties and antigenic targets rather than antibody class alone. Finally, we explore how the advances in our understanding of B cells may guide the development of more targeted immunomodulatory therapies that enhance atheroprotective B cell functions while limiting atherogenic responses.

## 1 Introduction

Despite substantial therapeutic progress in the past few decades, atherosclerotic cardiovascular disease (ASCVD) remains the greatest global burden of morbidity and mortality, accountable for approximately 18 million deaths each year [1, 2]. In parallel, improvements in healthcare and living conditions have led to an increase in life expectancy [3]. The United Nations estimates that by 2050, one in six people globally will be over the age of 65 [4]. As aging is a dominant risk factor for ASCVD, the societal and economic burden of age‐associated diseases such as atherosclerosis is expected to rise sharply. Atherosclerosis, the underlying pathology of most cardiovascular diseases (CVDs), has long been primarily regarded as a lipid‐driven process involving the progressive accumulation of cholesterol‐rich particles in the arterial wall. However, this view has advanced significantly and continues to evolve. A growing body of evidence now places chronic inflammation at the center of disease progression, from the earliest stages of fatty streak formation to the destabilization of advanced plaques [5, 6]. Even though acute cardiovascular events such as myocardial infarction and stroke typically occur at an advanced age, vascular changes already start early in life [7]. This process (summarized in Figure 4) is initiated by increased endothelial permeability in arteries exposed to shear stress, allowing subendothelial retention of circulating lipids, particularly low‐density lipoprotein (LDL) [8]. Within the intima, LDL particles are susceptible to various modifications, most notably oxidation, leading to the generation of oxidation‐specific epitopes (OSEs) that can be recognized as neo self‐antigens by the immune system [9]. These OSEs activate vascular cells, thereby triggering the upregulation of adhesion molecules and the production of chemokines and cytokines [10]. This pro‐inflammatory response facilitates the recruitment of circulating immune cells, particularly monocytes, into the vessel wall. Once recruited, monocyte‐derived macrophages phagocytose oxidized LDL (oxLDL), transforming into foam cells that further amplify local inflammation by attracting additional immune cells [11]. Over time, the accumulation of lipids and cellular debris from dying foam cells drives lesion progression and contributes to the formation of a necrotic core. Moreover, cellular debris serves as a source of OSEs, thereby further fueling the inflammatory response. In parallel, antigen‐presenting cells (APCs) present antigens to the adaptive immune system, predominantly in secondary lymphoid organs (SLOs) such as the spleen and draining lymph nodes, after which activated B and T cells can migrate to the site of inflammation. T cells constitute the largest proportion of immune cells infiltrating human atherosclerotic plaques, as shown by single‐cell RNA sequencing (scRNAseq) and mass cytometry analyses on carotid endarterectomy (CEA) samples [12, 13], and numerous studies have demonstrated that various T cell subsets, such as interferon gamma (IFNy)‐secreting T helper (Th) 1 cells, contribute to atherosclerosis development (reviewed in Ref. [14]). Although B cells only account for a small percentage of plaque‐resident immune cells [12, 13], they have been recognized as key modulators of atherosclerosis via both innate and adaptive immune mechanisms. B cells are predominantly observed in the adventitia [15] and perivascular adipose tissue (PVAT) [16], where they can form organized hubs together with T cells in artery tertiary lymphoid organs (ATLOs) [17, 18, 19, 20] and fat‐associated lymphoid clusters (FALCs) [16], respectively. Together, these microenvironments facilitate local B cell activation, thereby modulating plaque progression. In the past few decades, it has become apparent that these effects can be atherogenic or atheroprotective, which is largely determined by the specific B cell subset involved. In this review, we aim to provide a detailed and up‐to‐date overview on how different B cell subsets contribute to ASCVD, including recent insights on innate B cell populations producing IgM against OSEs (IgM^OSE^) and the emerging age‐associated B cells (ABCs).

## 2 B Cell Subsets, Development, and Activation

In the following section, we provide a broad overview of B cell subsets, development, and activation, providing the foundation for understanding their contributions to ASCVD.

### 2.1 B Cell Subsets

Different B cell subsets arise during development and have been defined by their origin, anatomical distribution, phenotypic markers, and functional properties. Most studies investigating these subsets have been performed in mice, revealing two broad subtypes: B1 and B2 cells. B1 cells (discussed in Section 4.1) predominantly originate from the fetal liver and are maintained by self‐renewal in the periphery [21]. They reside primarily in the peritoneal and pleural cavities, but upon T cell‐independent (TI) stimulation, they can migrate to antibody‐producing niches, including spleen, BM, and adipose tissue, where they can produce IgM [22, 23]. B2 cells account for the majority of B cells and originate from the BM and subsequently mature in the spleen, where they can differentiate into marginal zone (MZ) B cells (discussed in Section 4.1) or follicular (FO) B cells (discussed in Section 4.2). MZ B cells rapidly respond to blood‐borne antigens through T cell‐dependent (TD) and TI‐mediated mechanisms and produce both class‐switched and nonclass‐switched antibodies [24, 25]. More commonly, B2 cells differentiate into FO B cells, which primarily reside in the follicles of SLOs where they orchestrate TD‐mediated responses through germinal center reactions that give rise to both class‐switched and nonclass‐switched antibodies [26]. Another small B cell subset is regulatory B cells (Bregs); however, their ontogeny remains unclear. Bregs mediate their immunosuppressive function through the production of anti‐inflammatory cytokines such as IL‐10 and IL‐35 or via co‐inhibitory signals [27, 28, 29, 30].

### 2.2 B Cell Development

During B cell development, each B cell clone acquires a unique B cell receptor (BCR), which is a membrane‐bound immunoglobulin (Ig) that can bind antigens. This process is mediated by assembly of heavy and light chain polypeptides, which are generated through sequential gene recombination of variable (V), diversity (D), and joining (J) genes [31]. The heavy chain constant regions determine the isotype of the BCR, which can be IgM, IgD, IgA, IgE, and IgG, and determine the effector function of the antibody when secreted [32]. Due to the random nature of VDJ recombination, 55%–75% of immature B cells are estimated to express BCRs that recognize self‐antigens [33]. To avoid autoimmunity, autoreactive B cells binding self‐antigens with high avidity are subjected to central tolerance mechanisms including clonal deletion via apoptosis, silencing by attaining an anergic, unresponsive state, or BCR editing resulting in a different antigen specificity [34, 35, 36]. Immature B cells, of which 40% still moderately recognize self‐antigens [33], leave the BM as transitional B cells and can migrate to the spleen for the final stages of maturation [37]. During this phase, peripheral tolerance eliminates transitional B cells with high avidity BCRs to self‐antigens, thereby further reducing the frequency of autoreactive B cells [38]. When antigens fail to engage the BCR, either due to their absence or subthreshold concentration, B cells may remain in an immunologically ignorant state [39]. This allows for activation upon exposure of the antigen during, for example, inflammation or the emergence of neo self‐antigens. Once matured, naïve B cells home to serosal cavities or to SLOs, where antigen recognition can occur either in soluble form or presented by APCs such as subcapsular sinus macrophages or (follicular) dendritic cells (DCs) [40].

### 2.3 B Cell Activation

BCR stimulation by protein antigens initiates signaling cascades that drive antigen internalization and processing for major histocompatibility complex (MHC) class II presentation to cognate antigen‐specific Th cells [41]. Subsequent Th cell‐derived co‐stimulatory signals are essential for completing activation, enabling clonal expansion and differentiation of naïve B cells into antibody‐secreting cells and memory subsets. The canonical TD pathway for high‐affinity antibodies generation involves B cell activation within follicles of SLOs that differentiate into GC B cells and enter GC responses together with follicular Th cells (Tfh) and follicular dendritic cells (FDCs). In these specialized microenvironments, B cells undergo affinity maturation through repeated rounds of activation‐induced cytidine deaminase (AID)‐mediated somatic hypermutation (SMH) in the BCR variable region and antigen affinity‐driven selection. This process is supported by FDCs, which efficiently display antigens for BCR recognition. B cell clones compete for these antigens and subsequently present them to cognate antigen‐specific Tfh cells, which in turn provide necessary co‐stimulatory signals such as CD40 ligand (CD40L) and interleukin (IL)‐21 for affinity maturation [42, 43, 44]. Additionally, co‐stimulation can induce class‐switch recombination (CSR) to IgG, IgE, and IgA, which is influenced by the cytokine milieu in the GC. Following this process, GC B cells differentiate into proliferating plasmablasts, terminally differentiated short‐lived plasma cells (SLPCs), long‐lived plasma cells (LLPCs), which can migrate to the BM and provide long‐term humoral responses, or memory B cells, which circulate in a resting state and rapidly respond upon reexposure to the same antigen [45]. This GC response is regulated by follicular regulatory T (Tfr) cells, which can suppress Tfh and GC B cells, thereby preventing excessive inflammation [46].

During initial responses, TD activation often occurs GC‐independent or extrafollicularly, resulting in the fast generation of plasmablasts and SLPCs that rapidly provide the first wave of low‐affinity antibodies (reviewed in Ref. [47]). These antibodies are often assumed to remain of the IgM isotype but can also undergo CSR and SMH to give rise to high‐affinity IgG, IgA, or IgE antibodies [48, 49, 50]. Furthermore, these pathways might also give rise to LLPCs and memory B cells, as have recently been reviewed by Eisenbarth et al. [47] and Glaros et al. [51]. Notably, in autoimmune‐prone mouse models, autoantibody production has been reported to arise from extrafollicular (EF) responses, highlighting the importance of this pathway in autoimmunity [52, 53, 54].

**FIGURE 1:**
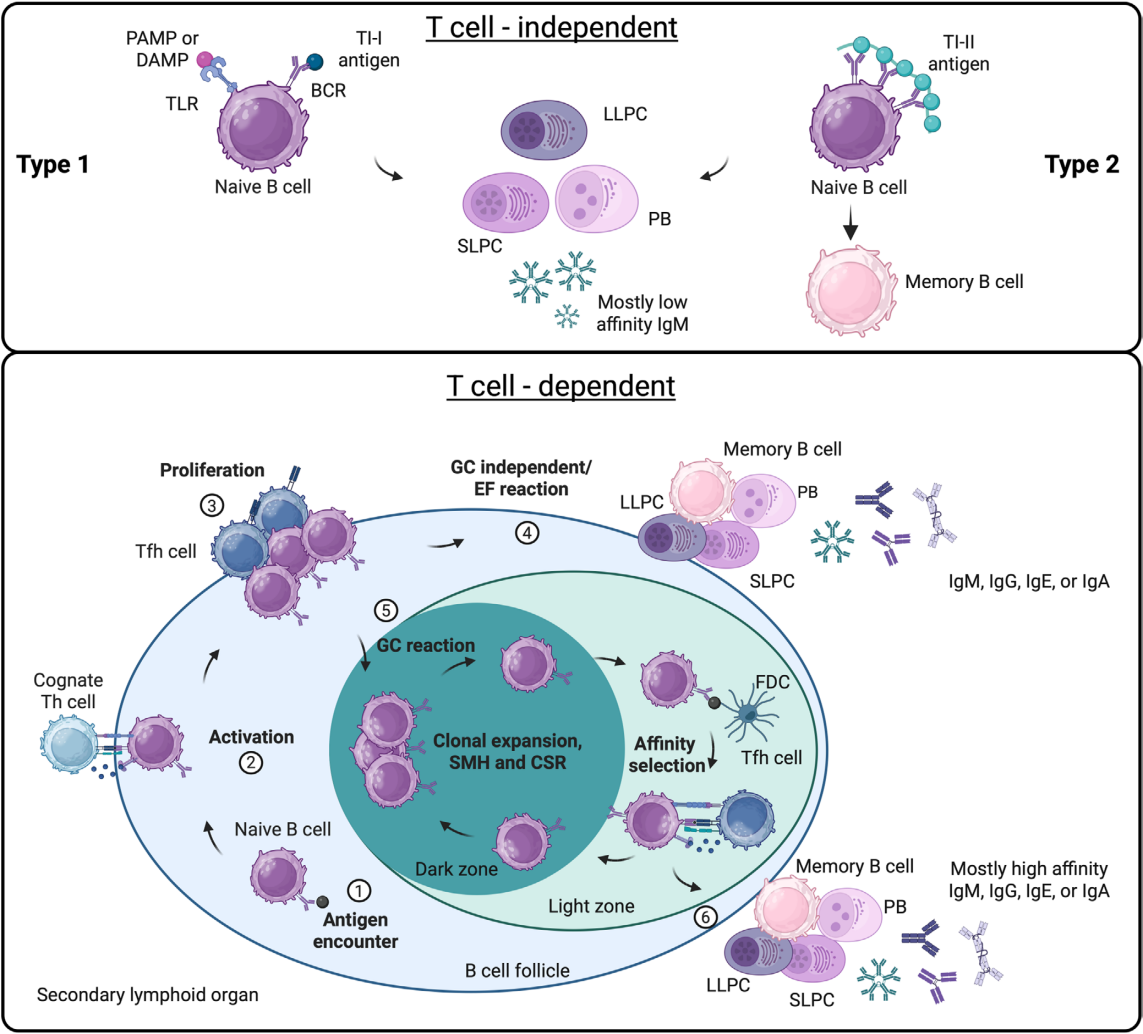
T cell‐independent and T cell‐dependent B cell activation pathways. B cells can be activated through T cell‐independent (TI) or T cell‐dependent (TD) pathways. Binding of TI type 1 (TI‐I) antigens (upper left) to the B cell receptor (BCR) of naïve B cells in combination with toll‐like receptor (TLR) stimulation via pathogen‐ or damage‐associated molecular patterns (PAMPs/DAMPs) drives B cell proliferation and differentiation into plasmablasts (PBs), short‐lived plasma cells (SLPCs), or long‐lived plasma cells (LLPCs) that predominantly secrete low‐affinity IgM. TI type 2 (TI‐II) antigens (upper right) activate B cells by crosslinking multiple BCRs, a pathway that can also generate memory B cells. In the TD pathway, naïve B cells in secondary lymphoid organs that encountered an antigen (1) migrate to the T‐B border of the B cell follicle where they can interact with cognate T helper (Th) cells (2), resulting in the activation and proliferation of the B and Th cells, the latter differentiating into T follicular helper (Tfh) cells (3). Activated B cells can then either follow a germinal center (GC) independent or extrafollicular (EF) pathway (4), rapidly generating PBs, SLPCs, LLPCs, and memory B cells that secrete low to high‐affinity IgM, IgG, IgE, or IgA, or they enter the GC reaction (5). Within the GC dark zone, GC B cells can undergo clonal expansion, somatic hypermutation (SMH), and class‐switch recombination (CSR). GC B cells entering the light zone can acquire antigen from follicular dendritic cells (FDCs) and subsequently present it to Tfh cells to receive signals required for affinity maturation. Positively selected GC B cells either reenter the dark zone or exit the GC to differentiate into PBs, SLPCs, or LLPCs predominantly producing high‐affinity antibodies, or into memory B cells (6).

B cells can also be activated in a TI manner in response to non‐proteinaceous antigens. TI type 1 (TI‐I) responses are driven through toll‐like receptor (TLR) stimulation, in addition to BCR activation, which recognize pathogen‐associated molecular patterns (PAMPs) and damage‐associated molecular patterns (DAMPs), such as OSEs and nucleic acids [55]. Furthermore, TI type 2 (TI‐II) antigens, for example polysaccharides, can cause BCR crosslinking and subsequent activation of naïve B cells due to their repetitive epitopes [56]. The resulting plasma cells from TI responses can be short‐ or long‐lived, and memory B cells have also been shown to be induced by TI‐II antigens, although these are phenotypically distinct from TD‐generated memory B cells [57, 58]. In addition, TI antibody class‐switching to IgA has been reported to occur at mucosal sites [59] and to IgG following vaccination with TI peptide‐ and adjuvant‐loaded liposomes [60]. These distinct B cell activation pathways have been summarized in Figure 1.

## 3 B Cell Functions in Atherosclerosis

### 3.1 Humoral Immunity

One of the primary effector functions through which B cells regulate atherosclerosis development is the secretion of various immunoglobulins (Figure 4). Their importance is supported by preclinical studies in which antibody production is impaired through plasma cell deletion, although with mixed outcomes. Centa et al. [61] reported that plasma cell deficiency induced by B cell‐specific deletion of the plasma cell differentiation factor *Prdm1* (encoding BLIMP1, B lymphocyte‐induced maturation protein 1) in mice (*Cd19* ^*cre*^ *Prdm1* ^*flox*^ *Apoe* ^*−/−*^) resulted in strongly reduced IgM and IgG titers, accompanied by smaller but less stable plaques, suggesting that the humoral response is pro‐atherogenic. In contrast, Sage et al. [62] observed increased lesion size in mice with a B cell‐specific X‐box binding protein 1 (*Xbp1*) deletion (*Cd79a* ^cre^ *Xbp1* ^*flox*^ BM cells transplanted into Ldl receptor‐deficient [*Ldlr* ^*−/−*^] mice), which is required for terminal plasma cell differentiation. Despite the limited presence of B cells and plasma cells within atherosclerotic plaques, systemically produced antibodies have been shown to abundantly deposit in the lesion via circulation [63, 64, 65, 66]. Here, they can recognize several (neo) self‐antigens, including oxidized lipoproteins and vascular wall‐associated antigens, as well as classical autoantigens such as double stranded DNA (extensively reviewed by Deroissart et al. [67]). However, the complete repertoire of atherosclerosis‐associated antigens and their corresponding autoantibody responses remain to be fully characterized. Nevertheless, in the last few decades it has become clear that these antibody responses can ameliorate or aggravate disease progression, depending on the antibody isotype and their antigen specificity.

#### 3.1.1 IgM

**FIGURE 2:**
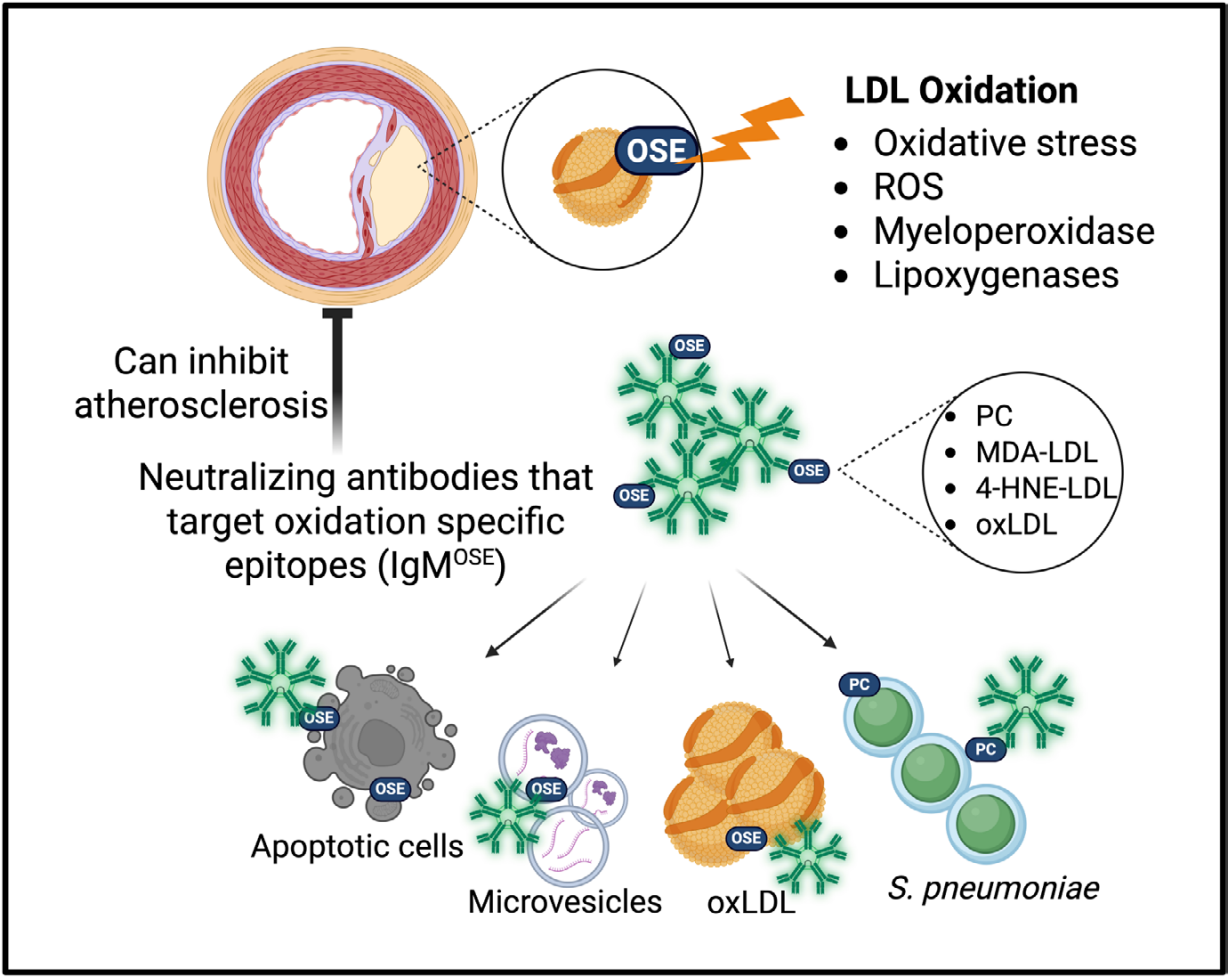
Schematic of low‐density lipoprotein oxidation and IgM^OSE^ neutralization. Atherosclerosis is a chronic inflammatory disease that facilitates the retention of low‐density lipoprotein (LDL) particles in the subendothelial wall where the retained LDL particles are highly susceptible to modification, particularly oxidation through both enzymatic (myeloperoxidase and lipoxygenases) and nonenzymatic oxidative stress, reactive oxygen species (ROS) processes, leading to the generation of oxidation‐specific epitopes (OSE). These OSEs can be found not only on LDL but also on apoptotic cells, microvesicles, and share molecular mimicry of phosphorylcholine (PC) epitopes found on *Streptococcus pneumoniae* ( *S. pneumoniae* ). MDA, malondialdehyde; 4‐HNE, 4‐hydroxynonenal.

IgM is a pentameric immunoglobulin that has been linked to atherosclerosis in both mice and humans. IgM functions in many aspects of atherogenesis, including promoting the clearance of dead cells, neutralizing pro‐inflammatory atherosclerotic antigens, inhibiting foam cell formation, and reducing the thrombogenic activity of extracellular vesicles released by apoptotic or activated cells [68, 69, 70, 71, 72, 73, 74, 75, 76]. A large amount of secreted IgM in mice is considered to be natural IgM that is generated prior to any antigen exposure, composed primarily of VDJ gene segments that have a higher propensity for autoreactive antigens [77, 78, 79]. However, IgM levels can be increased upon antigen exposure, allowing B cells to mount antigen‐specific responses and become memory B cells [80, 81, 82]. The first natural IgM shown to be linked to ASCVD was E06. E06 is an autoantibody with the T15 idiotype that recognizes conserved epitopes of phosphorylcholine (PC) on oxidized phospholipids and apoptotic cells [78, 83, 84, 85, 86] (Figure 2). Immunization of hyperlipidemic animals with oxLDL and pneumococcal immunogen increased serum levels of oxLDL‐specific IgM antibodies, including E06, and reduced progression of atherosclerosis [78, 84, 85, 87, 88, 89]. These studies emphasize the cross‐reactivity of natural IgM antibodies and their broad specificity in recognizing self‐antigen in response to both pathogen exposure and inflammation‐generated oxidative stress. IgM^OSE^ have also been implicated in human atherosclerotic disease. Plasma levels of IgM^OSE^ in humans inversely correlate with angiographically determined coronary artery disease (CAD) [72, 90] and with an increased risk of CAD events [74, 91]. Additional studies corroborated these findings, showing that human IgM antibodies targeting malondialdehyde‐modified LDL (MDA‐LDL), an OSE antigen, were negatively associated with CVD [71]. Later sections of the review will highlight the regulation of IgM^OSE^ production by these innate B cell classes in both mice and humans and their impacts on atherosclerosis. We focus these sections of the review on IgM^OSE^, while acknowledging that IgM against other atherosclerotic antigen targets have been shown to associate with reduced CVD [73, 92, 93, 94, 95, 96, 97].

#### 3.1.2 IgG

IgG is the most abundant immunoglobulin in circulation and can be divided into four subclasses. In mice, these include IgG1, IgG2b, IgG2a (or IgG2c, depending on the mouse strain), and IgG3, whereas in humans they are classified as IgG1–4, such that human IgG1 corresponds mostly to mouse IgG2a/c, human IgG2 to mouse IgG3, human IgG3 to mouse IgG2b, and human IgG4 is equivalent to IgG1 in mice [67]. Notably, human and murine IgGs and their receptors display considerable differences, which hinder straightforward comparisons between the two species [98]. IgGs can exert their functions through various mechanisms (reviewed in Ref. [67]), including neutralization of target antigens and the formation of immune complexes, which may be atheroprotective through clearance after opsonization, with or without recruitment of the complement system, or can mediate cytotoxicity in a cell‐dependent or complement‐dependent manner. Furthermore, IgGs can cause cellular activation or inhibition through binding of the terminal part of the constant heavy region to different Fc γ receptors (FcγRs) present on effector cells. Engagement of activating FcγRs on endothelial cells, vascular smooth muscle cells, and monocytes/macrophages elicits diverse cellular responses that can promote the development of CVD [99]. Given these broad range of effector functions, it might not be surprising that studies investigating the involvement of IgG in human CVD have generated conflicting findings. One study by Khamis et al. [100] showed that higher total IgG serum levels were negatively associated with the risk of cardiovascular events in patients with hypertension, while other studies reported positive associations between IgG and cardiac death and myocardial infarction in men with hyperlipidemia [101] or with increased ASCVD risk in middle‐aged and older individuals [102]. Thus, the role of IgG in CVD is likely not determined by its overall levels, but also by its antigen specificity, with atherogenic dyslipidemia pushing the antigen specificity of the IgG repertoire toward OSEs and other neo self‐antigens. The first evidence for this was provided by Tay et al. [65] who demonstrated that transfer of purified polyclonal IgG from atherosclerotic *Apoe* ^*−/−*^ mice into *Ldlr* ^*−/−*^ mice with reduced endogenous IgG through plasma cell deficiency resulted in increased IgG deposition in the atherosclerotic lesions and enhanced plaque formation compared to administration of IgG derived from non‐atherosclerotic wild‐type mice. In line with this, the IgG1 antigen‐repertoire of high‐fat diet‐fed *Apoe* ^*−/−*^ mice is enriched in self‐antigens compared to wild‐type controls [103] and increased antinuclear IgGs and IgM are produced in *Apoe* ^*−/−*^ mice on high‐fat diet compared to normal chow diet [104]. Nevertheless, in humans, levels of antigen‐specific IgGs to OSEs or in the form of immune complexes have yielded evidence for positive associations [94, 105, 106], no associations [74, 107, 108, 109] or even a negative association with CVD [92]. In the majority of these studies, only total antigen‐specific IgGs were assessed, without distinguishing between IgG subclasses. The different IgG subclasses have different affinities for the various activating and inhibiting receptors and combinations of receptors are expressed on most cells [110]. However, the role of individual IgG subclasses in atherosclerosis development has not yet been extensively studied in CVD patients and is still underexplored in atherosclerosis mouse models. Different IgG subclasses are produced depending on the prevailing cytokine milieu. For example, Th2 cytokine IL‐4 can induce class‐switching to IgG1 or IgE in mice, while Th1 cytokine IFNy induces switching to IgG2a/c and IgG3 [111]. Alternatively, the presence of transforming growth factor‐β (TGF‐β) results in switching to IgG2b and IgA [112]. Additionally, the switching is strongly influenced by the type of antigen, with TI antigens inducing mostly IgG2b and IgG3 production, whereas IgG1 and IgG2a/c are considered to be part of the TD response [113].

##### 3.1.2.1 Murine IgG1/Human IgG4

In several inflammatory autoimmune disease models, IgG1 deficiency has been shown to result in severe autoimmune conditions, suggesting an overall anti‐inflammatory role for IgG1 antibodies [114, 115]. IgG1 has relatively low affinity to most activating FcγRs, poorly activates the complement system, and is more likely to neutralize proatherogenic antigens, thereby making it potentially atheroprotective. Consistent with this, immunization of *Ldlr* ^*−/−*^ mice with MAA‐MSA (malondialdehyde‐acetaldehyde‐modified mouse serum albumin), which was demonstrated to be atheroprotective, mounted a strong MAA‐specific IgG1 response [116]. In addition, plasma isolated from these mice blocked the uptake of MAA‐LDL by macrophages in vitro, although this inhibition could in part have been mediated by elevated titers of natural IgM E06, which also recognizes MAA‐LDL. In contrast, Zhang et al. [117] reported that vaccination with recombinant histone 2B (H2B) significantly increased atherosclerotic lesions in *Apoe* ^*−/−*^ mice and was accompanied by high levels of H2B‐specific IgG1, thereby suggesting a proatherogenic role for H2B‐specific IgG1. Another mechanism through which IgG1 could confer an atheroprotective effect is by binding the inhibitory receptor FcγRIIb, present on various immune cells, which it does with relatively high affinity compared with its binding to its only activating receptor, FcγRIII [110, 118]. Even though deficiency of FcγRIIb is associated with autoimmunity [119], studies investigating the role of this receptor in atherosclerosis have generated conflicting results, showing cell type and sex‐specific effects [120, 121, 122, 123, 124, 125].

Whereas IgG1 is the most abundant IgG in mice, IgG4, the mouse IgG1 equivalent in humans, represents only a minor fraction. Despite its lower abundance, human IgG4 shares functional similarities with murine IgG1, including anti‐inflammatory properties such as low affinity toward activating FcγRs [126]. Furthermore, its induction is generally associated with immune tolerance and limiting inflammation. However, IgG4 has also been shown to drive certain autoimmune diseases [127, 128, 129] and cardiovascular conditions such as inflammatory abdominal aortic aneurysm and coronary periarteritis [130, 131]. Serum concentrations of IgG4 have been shown to be increased in patients with CAD compared with healthy controls [117], and IgG4 was significantly associated with noncalcified and low‐density plaques in CAD patients, characteristics associated with plaque vulnerability [132]. Additionally, a recent study of Lai et al. [133] using scRNAseq and spatial transcriptomics on human carotid plaques observed significantly higher gene expression levels of IgGs, including IgG4, in plasma cells of symptomatic patients who experienced cerebrovascular events compared to asymptomatic patients. Still, it remains to be investigated whether increased IgG4 levels contribute to atherosclerosis progression or reflect a compensatory mechanism to dampen the ongoing inflammation.

##### 3.1.2.2 Murine IgG2b/Human IgG3

IgG2b class‐switching is induced by TGF‐β, which is one of the key cytokines produced by atheroprotective regulatory T cells (Tregs) [134, 135], suggesting a potential atheroprotective role for IgG2b. Supporting this, the passive immunization of *Ldlr* ^−/−^ mice with mouse IgG2b specific for electronegative LDL (LDL(−)) significantly reduced lesion development compared to irrelevant monoclonal IgG‐treated control mice and was paired with lower levels of circulating free LDL(−) [136]. Furthermore, Lorenzo et al. [97] recently showed that IgG2b antibodies specific for the self‐antigen aldehyde dehydrogenase 4 family member A1 (ALDH4A1) can reduce circulating free cholesterol and LDL and inhibit plaque progression. The reduction in circulating LDL particles observed in these studies could reflect clearance through neutralization mediated by the IgG2b antibodies, or via opsonization pathways involving complement or FcγRs, all of which are effector mechanisms of this subclass [67]. Nevertheless, the exact mechanisms underlying IgG2b's role in atherosclerosis require further investigation.

Similar to mouse IgG2b, human IgG3 is a potent activator of the complement system and induces effector functions through FcγRs, making it possibly the closest equivalent. However, human IgG3 is unique because of its unusually long hinge region, which confers increased flexibility and likely enhances its ability to activate complement via C1q binding [137, 138]. Being one of the main responders against viral infections, IgG3 is a potent pro‐inflammatory antibody and could therefore be proatherogenic. However, this subclass has not been thoroughly studied in clinical cohorts of patients with ASCVD. Nevertheless, in vitro recombinant humanized IgG3 antibodies specific for collagen IV, which exhibit high affinity for activating FcyRIA, were demonstrated to enhance oxLDL‐induced monocyte chemoattractant protein 1 (MCP‐1) release in CD14^+^ monocytes and promote differentiation into pro‐inflammatory classical monocytes [139].

##### 3.1.2.3 Murine IgG2a/Human IgG1

Functionally, IgG2a/c resembles IgG2b in its ability to activate the complement system and engage with FcγRs [98, 140]. Yet, IgG2a/c displays a relatively high FcγR activation‐to‐inhibition ratio, with a bias toward binding FcγRIV rather than FcγRIIb, thereby shifting the balance toward immune activation [110, 141]. In line with this, atherosclerotic DCs and macrophages express FcγRIV, and in vitro IFNγ‐stimulated bone‐marrow‐derived DCs upregulate FcγRIV and secrete elevated levels of tumor necrosis factor (TNF) when exposed to IgG2c‐coated beads, supporting a pro‐atherogenic role for IgG2c [124]. Consistently, Innate Response Activator (IRA) B cells are thought to mediate their atherogenic functions through GM‐CSF‐mediated expansion of classic DCs, which drives IFNγ‐producing atherogenic Th1 cell differentiation [142]. Subsequent interaction between these Th1 cells and B cells can stimulate class‐switching from IgG1 to IgG2a. Another way through which IgG2a may exert its function is via complement‐dependent cytotoxicity. One example of this mechanism is IgG2a antibodies targeting endothelial heat‐shock protein 60 (HSP60), a cell‐surface protein triggered by stress factors such as hyperlipidemia or hypertension. Foteinos et al. [143] showed in vitro that the anti‐HSP60 IgG2a antibody has a cytotoxic effect on endothelial cells in the presence of complement. They also demonstrated that its administration into *Apoe* ^*−/−*^ mice results in increased lesion development compared to control mice [143].

Murine IgG2a/c closely resembles human IgG1 in both pharmacokinetics and Fc‐mediated effector functions, although human IgG1 has a higher relative abundance [137, 144]. Nevertheless, several epidemiological studies have indicated a possible atheroprotective function for IgG1 antibodies. For instance, Samal et al. [145] reported an association between low levels of anti‐PC IgG1 (below the 33^rd^ percentile) and an increased risk of CVD, including stroke and myocardial infarction, in 60 year olds. In contrast, high levels (above the 90^th^ percentile) were significantly associated with protection against stroke [145]. Furthermore, high anti‐MDA IgG1 levels (above the 66^th^ percentile) were shown to be associated with having approximately half the risk of developing CVD in the same cohort [109]. In line with these findings, a chimeric monoclonal anti‐PC IgG1 antibody (human IgG1 Fc and mouse T15/E06 Fab), which binds PC, oxLDL, and apoptotic cells, inhibited oxLDL uptake by human macrophages in vitro and reduced subsequent C‐C motif chemokine ligand (CCL) 2 production, thereby supporting an antigen‐neutralizing role for human IgG1. In vivo, this antibody was found to exert an atheroprotective role by decreasing neointima formation in a femoral artery cuff model in Apo3*Leiden mice [146].

##### 3.1.2.4 Murine IgG3/Human IgG2

In contrast to the other IgG subclasses, murine IgG3 does not interact with FcγRs [118], but can efficiently activate the complement system in response to TI antigens such as carbohydrates [147]. Its tendency to self‐aggregate can generate bivalent antibodies capable of multivalent binding with high functional affinity, enabling recognition of epitopes that are variably spaced on pathogen surfaces [148, 149]. This cooperative Fc–Fc interaction also enhances complement activation, giving IgG3 a functional polyvalency for C1q binding that resembles IgM. As a result, IgG3 could facilitate phagocytic clearance of opsonized particles via C1qR or complement‐dependent cytotoxicity, though it remains to be investigated whether these mechanisms contribute to atherosclerosis development.

In humans, IgG2 antibodies play a key role in defense against encapsulated bacteria but are relatively poor activators of the complement system compared to IgG1 and IgG3 and have low binding affinity for FcγRs [98, 118]. High IgG2 antibody levels specific for PC and MDA have been associated with protection against CVD, although these associations were weaker than those of IgG1 [109, 145]. In contrast, high levels of anti‐MDA IgG2 (above 75th percentile) were associated with an increased chance of MI/angina in women, but not in men [109], making it challenging to define IgG2's role in CVD.

#### 3.1.3 IgA

IgA antibodies are primarily secreted at mucosal surfaces, such as the respiratory and digestive tracts, where they can neutralize antigens associated with inhaled and ingested pathogens. Their role in atherosclerosis is not well understood and is difficult to study in mice due to low translatability to humans [150]. While humans possess two IgA subclasses, namely IgA1 (usually monomeric) and IgA2 (usually dimeric), mice have one IgA subclass. Moreover, humans have two Fc receptors (Fcα/μR and FcαRI) through which IgA can exert its functions, but mice lack FcαRI. This receptor is expressed on various cells, including DCs and macrophages, and binding with IgA immune complexes can result in several responses, such as phagocytosis, antigen presentation, and cytokine release [151, 152, 153]. To this end, IgA remains largely unexplored in atherosclerotic mouse models. Nevertheless, IgA levels have been reported to be slightly increased in *Apoe* ^*−/−*^ mice on a high‐fat diet compared to C57BL/6J wild‐type mice [103].

In humans, several epidemiological studies have reported that high total serum IgA levels are associated with CVD risk [101, 102, 154, 155] as well as high levels of anti‐PC IgA antibodies [156]. However, this was challenged by Akhi et al. [157], who showed that individuals with higher carotid intima‐media thickness, an indicator of atherosclerosis progression, had lower levels of total and anti‐PC IgA. Additionally, this study reported an inverse association between oral microbiome diversity and carotid intima‐media thickness in male participants, alongside a correlation between atherosclerosis‐associated oral bacteria and anti‐PC IgA levels, implicating a role for oral mucosal immunity in atherosclerosis development. These findings align with growing evidence linking the gut microbiome to CVD [158].

#### 3.1.4 IgE

IgE is generally considered proatherogenic, as *Apoe* ^*−/−*^ mice lacking IgE or its receptor FcεRI exhibit reduced lesion formation [159, 160]. While FcεRI is primarily expressed on basophils, eosinophils, and mast cells, it can also be found on macrophages [160, 161]. Due to its high affinity, IgE can bind to FcεRI even in the absence of an antigen, and subsequent antigen engagement triggers cellular activation. In atherosclerosis, this activation can contribute to plaque development through multiple mechanisms. Mast cells promote plaque destabilization by releasing proteases, histamine, and cytokines such as IL‐6 and IFNγ [162, 163, 164]. Supporting this, IgE‐mediated systemic mast cell activation in B cell‐deficient *μMT* ^*−/−*^ *Apoe* ^*−/−*^ mice was shown to increase plaque size [165]. Similarly, IgM‐deficient mice exhibit elevated IgE levels, which lead to increased mast cell activation and plaque development, a phenotype that can be reversed by anti‐IgE treatment [166]. Beyond mast cells, IgE can act on FcεRI‐expressing macrophages, inducing their activation, polarization, and foam cell formation, thereby amplifying local inflammation in the plaque [159, 160].

Multiple studies link elevated IgE levels to human CVD, and the expression of both IgE and FcεRI in atherosclerotic carotid artery lesions points to a potential role for IgE in the pathogenesis of atherosclerosis [101, 159, 167, 168, 169, 170, 171]. Nevertheless, the precise antigenic triggers of IgE responses in atherosclerosis have yet to be defined. One candidate antigen is mammalian oligosaccharide galactose‐α‐1,3‐galactose (α‐Gal), as anti‐α‐Gal IgE was associated with atheroma burden and plaque instability in subjects under the age of 65 [172]. Furthermore, Keet et al. [173] recently found that individuals sensitized to at least one food, particularly cow's milk, had an increased risk of cardiovascular mortality.

### 3.2 Antibody‐Independent Functions

In addition to their role in humoral immunity, B cells can act as APCs for naïve Th cells via MHC‐II expression, thereby driving T activation and Tfh differentiation [174]. Furthermore, antigen presentation by B cells to Tfh cells is essential for effective GC responses [45]. The role of B cell‐specific MHC‐II in atherosclerosis, recently reviewed in Ref. [175], is not completely elucidated yet, but is thought to be pro‐atherogenic as its deficiency was shown to reduce atherosclerosis development in a BM chimeric approach by Tay et al. [65]. In addition to antigen presentation, B cells express a range of immune checkpoint molecules and secrete various cytokines and chemokines that enable crosstalk with other (immune) cells, primarily T cells. Upon priming by Th1 or Th2 cells, murine B cells have been shown to release IFNγ, IL‐12, and TNFα or IL‐2, IL‐4, TNFα, and IL‐6, respectively, as so‐called effector B cells [176]. These cytokines have the potential to promote atherosclerosis development (reviewed in Ref. [177]). However, by secreting anti‐inflammatory cytokines such as IL‐10 and TGF‐β, B cells can also mediate atheroprotective functions [178, 179, 180]. Additionally, tertiary lymphoid organ formation is dependent on lymphotoxin α1β2 production by B cells [181]. The exact secretory and immune checkpoint expression profile of B cells is highly subset‐dependent and will therefore be discussed in more detail in the following sections.

## 4 B Cell Subsets and Atherosclerosis

Early murine work performed by Caligiuri et al. [86] suggested that B cells are protective against hyperlipidemia‐induced atherosclerosis, as adoptive transfer of splenic B cells rescued splenectomy‐induced lesion progression in *Apoe* ^*−/−*^. Likewise, BM transplantation from B cell‐deficient μMT mice into lethally irradiated *Ldlr* ^*−/−*^ recipients resulted in increased plaque development compared to controls [182]. In contrast, Tay et al. reported that μMT *Apoe* ^*−/−*^ mice have decreased atherosclerotic burden relative to controls [183] and mature B cell depletion using CD20‐specific antibodies reduced atherosclerosis in *Apoe* ^*−/−*^ and *Ldlr* ^*−/−*^ mice [184], pointing toward a pro‐atherogenic role for B cells. These seemingly contradictory findings were later clarified through more detailed studies of B cell subsets in the context of atherosclerosis development.

In the following sections of this review, the specific contribution of major B cell subsets to atherosclerosis development will be discussed in greater detail and is summarized in Figure 4.

### 4.1 B1 and Marginal Zone B Cells

#### 4.1.1 Murine B1 Cells and IgM^OSE^

B1 cells characterized in the Herzenberg's laboratory as Ly‐1^+^ B cells [185, 186, 187], constitute the first and most primitive wave of B cells in the layered immune system [188], so termed B1 or B1a cells. An additional wave is generated by B1b cells, a Ly‐1^−^ sister population, which are similar in some respects to B1a cells but, unlike B1a cells, they do not express CD5, and they exhibit greater SMH, memory, and function of the BCR [77, 81, 189, 190].

A unique feature of B1 cells (both B1a and B1b) is their ability to produce natural IgM antibodies with broad antigenic specificities, including oxidatively modified phospholipids. The identification of oxLDL‐specific IgM antibodies with the T15 idiotype linked B1 cells to atherosclerosis, as B1a cells are the primary producers of T15 clonotype antibodies [191]. As T15/E06 has a restricted variable region with minimal N additions [192, 193, 194], B1a cells were thought to be the main producers of natural IgM in vivo. Chou et al. [195] subsequently demonstrated that B1 cell‐derived IgM (~30%) could target various OSEs (PC, copper‐oxidized LDL [CuOx‐LDL], 4‐hydroxynonenal LDL [4‐HNE‐LDL], MDA‐LDL), but not native LDL. As repertoire sequencing of B1 cells showed more N additions in B1b than B1a cells [77, 196], this suggested a potential unique role for the B1b cell. Haas et al. [81] reported on an important “division of labor” within the B1 compartment using a murine model of *Streptococcus pneumoniae* ( *S. pneumoniae* ) infection. They found that while B1a cells secreted natural antibodies that protected against acute antigen exposure, B1b cells generated long‐lasting IgM memory in a TI manner to support survival [81]. Whether B1b cells produce atheroprotective IgM^OSE^ and attenuated atherosclerosis was first shown by Rosenfeld et al. [197]. They demonstrated that B1a cells produced higher levels of IgM^OSE^ in vitro following lipopolysaccharide (LPS) stimulation than B1b cells. However, B1b cells produced more IgM^OSE^ than B1a cells in vivo after adoptive transfer into *Rag* ^*−/−*^ *Apoe* ^*−/−*^ mice. This finding is consistent with data demonstrating that B1a and B1b cells have unique BCR repertoires at homeostasis [81, 190]. In the context of hyperlipidemia, Srikakulapu et al. [77] found that injection of B1b cells, compared to B1a cells, resulted in greater migration to the spleen and higher levels of local and global IgM. Moreover, this study showed that B1b cells undergo more N additions and SMH, consistent with their capacity to respond to antigens [77]. B1a cell repertoires; however, have fewer mutated sequences and are characterized by predictable VH‐region usage [77]. Despite differences in BCR repertoires and antigenic response capacity, adoptive transfer of both B1a [66] and B1b [197] cells has been shown to attenuate hyperlipidemia‐induced atherosclerosis in mice.

##### 4.1.1.1 BCR and TLR Regulation of IgM^OSE^ Production by Murine B1 Cells

BCR and TLR signaling work both independently and in concert to regulate IgM^OSE^ antibody responses in innate B cells [198, 199, 200]. BCR signaling is imperative for the development and function of B cells; however, it is not well understood how BCR signaling regulates IgM production in the context of atherosclerosis. Rather, studies have shown that the high antigenic environment of atherosclerosis drives B1 cells to produce IgM^OSE^ [199]. Secreted IgM can act as a negative regulator of BCR signaling by binding to OSE and preventing BCR activation [199]. Building on the idea that strong BCR signaling is important for B1 cell development, Gruber et al. focused on the effects of manipulating Sialic Acid‐Binding Immunoglobulin‐like Lectin G (Siglec G), a known inhibitor of BCR signaling [201], on B1 cells in atherosclerosis [202]. This study demonstrated that mice deficient in Siglec G had increased B1 cell frequency, increased IgM^OSE^, and reduced diet‐induced atherosclerosis [202]. This further supports that balanced regulation of BCR signaling is key in mediating atheroprotective responses by B1 cells.

Recent work has focused on the role of CD19 in innate B cell function, as CD19 is not only a B cell lineage marker in both mice and humans, but also a co‐receptor for BCR signal transduction [203, 204]. Zhao et al. [205] analyzed the effects of *Cd19* ^*Cre/+*^ *Ldlr* ^*−/−*^ mice on innate B cells, finding that CD19 haploinsufficiency reduced diet‐induced atherosclerosis, increased the proliferative and immunosuppressive capacity of B1 cells compared to WT littermate controls, and increased secreted IgM within the PVAT. These data not only support the argument that BCR signaling is an important regulator of innate B cell atheroprotective IgM production, but also highlight the importance of the utilization of *Cd19* ^*Cre/+*^ controls when using this Cre mouse to investigate the role of B cell‐related genes in atherosclerosis.

Given the molecular mimicry of conserved bacterial epitopes with those found on atherosclerotic antigens [89, 206], it follows that the TLR signaling pathway is relevant to IgM^OSE^ antibody production and atheroprotection. In fact, B1a cells are particularly selective for TLR‐mediated stimulation to induce antibody production, as they express CD5 (unlike B1b cells), a dominant‐negative regulator of BCR antigenic signaling [207, 208, 209]. This is functionally emphasized by the finding that in vitro TLR4 stimulation of B1a cells produced higher levels of IgM^OSE^ than B1b cells [197]. Yet, global TLR deficiencies have been shown to modulate atherosclerosis differently depending on the TLR involved [210]. Compared to mice deficient in other TLR receptors, the adoptive transfer of TLR4‐deficient B1a cells into hyperlipidemic mice resulted in reduced production of IgM^OSE^ antibodies and increased diet‐induced atherosclerosis compared to controls [211] emphasizing the importance of TLR4 signaling in mediating B1a‐associated IgM^OSE^ production. TLR9, however, has been shown to limit IgM production and push B1 cells toward a more tolerogenic phenotype through increasing secretion of IL‐10 [212, 213]. Recent work by Baumgarth and colleagues provides a novel role for TLR signaling in altering the BCR signalosome, thereby enhancing B1 cell antibody responses to infection [200]. Taken together, these data implicate the importance of TLR signaling in mediating IgM^OSE^ production by innate B cell subsets and emphasize the need for further characterization of the roles of specific TLR receptors in innate B cells, as well as cooperative signaling with other B cell‐associated receptors.

##### 4.1.1.2 Chemokine Receptors

Specific chemokine receptors promote B1 cell trafficking to antibody‐producing niches, thereby increasing IgM^OSE^ production. While both B1a and B1b cells require CXCR5 and CCR6 to traffic to the BM and the spleen [214, 215], B1a cells uniquely require CXCR4 expression to traffic to the BM and subsequently produce IgM^OSE^ [23]. B1b cells also have unique chemokine receptor expression, with higher baseline expression of CCR6 in the peritoneal cavity, suggesting that these cells may more readily traffic to the spleen [77]. Moreover, CCR6 expression has been associated with increased B1 cell trafficking to adipose tissue [214]. Visceral adipose tissue (VAT) depots, specifically the omentum and PVAT, are other sites to which B1 cells traffic. Within the VAT, B1 cells can be found in FALCs, along with other innate and adaptive immune cells and VAT stromal cells. There, these cells promote B1 cell proliferation and antibody production, and they produce anti‐inflammatory cytokines such as IL‐10 (summarized in Figure 4) [16, 214, 216, 217, 218].

#### 4.1.2 B1 Cytokine Production

The atheroprotective role of B1 cells may not be limited to antibody production, as B1 cells also produce cytokines [86]. Early studies showed that both murine B1 cells and human putative B1 cells produced IL‐10 following antigenic stimulation [219, 220, 221]. B1 IL‐10 production has been shown to modulate macrophage activity, shifting macrophages toward a more anti‐inflammatory phenotype [222, 223]. Grimm et al. [223] demonstrated that macrophages with genetic downregulation of inflammatory pathways promoted the production of IgM^OSE^, increased local IL‐10 production by B1 cells, and reduced diet‐induced atherosclerosis. Beyond IL‐10, a recent study by Kang et al. [224] demonstrated that B1a cells can secrete IL‐27 containing exosomes to suppress inflammatory T cell responses to infection. IL‐27 has been established as an important yet controversial cytokine in atherosclerosis: some groups have shown that IL‐27 ameliorates atherosclerosis in mice, while others have shown that IL‐27 in plasma associates with increased CAD burden in humans [225, 226, 227, 228, 229, 230, 231]. In addition to regulating anti‐inflammatory cytokines, B1 cells have been shown to modulate T cell‐mediated immune responses by producing IFNγ, TNFα, IL‐2, and IL‐4 [232]. While the role of these cytokines in influencing T cell responses in atherosclerosis has been well studied and reviewed [14], it is uncertain how specifically B1 cell production of these cytokines might influence atherosclerosis progression. Moreover, Hilgendorf et al. [142] reported that B1a‐derived IRA B cells can produce GM‐CSF, potentially shifting to a more pro‐inflammatory type 1 immune response and worsening atherosclerosis. Despite current findings, the mechanisms by which innate B cell–mediated cytokine production contributes to atherosclerosis progression remain poorly understood.

#### 4.1.3 Murine Marginal Zone B Cells

Less is known about the role of MZ B cells (CD19^+^ B220^hi^ CD93^−^ CD21^hi^ CD23^low^ CD1d^hi^) in murine atherosclerosis than that of B1 cells. Early evidence that splenectomized mice develop increased atherosclerosis suggested a protective role for MZ B cells, given their localization in the marginal zone of the spleen [86, 182]. Subsequent work by Oliver et al. demonstrated that MZ B cells produced higher levels of IgM and IgG3 in response to LPS stimulation compared to FO B cells [233], suggesting a more innate‐like response to TI antigens, similar to that of B1 cells [234]. Aligning with this more innate‐like response to antigenic stimulation by MZ B cells, TLR stimulation, particularly through TLR4 and 9, has also been shown to be important in both MZ B cell TI and TD antibody responses [235, 236]. In fact, Genestier et al. [237] showed that both B1 and MZ B cells are primed to differentiate into IgM‐producing plasma cells following TLR ligation with TI antigens. Moreover, TLR signaling has been shown to play a role in innate B cell migration, specifically the migration of MZ B cells into follicles and of B1 cells from their homeostatic serosal spaces to antibody‐producing niches [238]. However, the deeper mechanisms by which specific TLRs regulate IgM^OSE^ production by MZ B cells in the context of atherosclerosis remain unclear.

One of the earliest analyses of the marginal zone compartment in hyperlipidemic mice by Soh et al. [239] revealed that the number of MZ B cells significantly increases during hypercholesterolemia. A functional link of this expansion of MZ B cells in hyperlipidemia was later established by Nus et al. [240], who found that the transfer of MZ B cell‐deficient BM into irradiated *Ldlr* ^*−/−*^ mice resulted in increased diet‐induced atherosclerosis through Tfh cell modulation. Moreover, studies have supported that in hyperlipidemic mice, MZ B cells have strong levels of BCR signaling, as measured by Nur77, a transcription factor increased upon BCR engagement [241]. Most recently, Harrison et al. [242] showed that MZ B cells regulate IgM^OSE^ production in a Tfh‐dependent manner. Notably, this study was the first to identify the role of MZ B cells in the production of IgM^OSE^ antibodies, specifically anti‐PC (E06/T15) antibodies, in hyperlipidemic mice. Collectively, these studies establish an emerging role for MZ B cells in modulating atherogenesis through IgM^OSE^ production.

#### 4.1.4 Human B1 and Marginal Zone B Cells

Compared to mice, much less is known about the B cells that produce IgM^OSE^ in humans. This is driven in part by limited access to healthy human serosal cavities, which makes complete characterization of human B1 cells challenging. Additionally, surface markers that define B cell subtypes in humans differ from those in mice. To address this, Griffin et al. [243] tested human B cell populations for three functional characteristics of murine B1 cells as an approach to determine the human B1 equivalent: TI and spontaneous production of IgM, tonic intracellular signaling, and efficient T cell stimulation to screen B cell populations in human umbilical cord and adult peripheral blood. Fulfilling all three characteristics, CD20^+^ CD27^+^ CD43^+^ B cells were suggested to be putative human B1 cells [243]. These putative B1 cells were found to bind phospholipid and DNA epitopes linked to autoimmunity [73, 243]. However, controversy ensued, and follow‐on analysis raised the concern that CD20^+^ CD27^+^ CD43^+^ B cells were actually preplasmablasts [244]. A subsequent scRNAseq of human prenatal tissue identified CD5^+^ CD27^+^ CD43^+^ cells as putative human prenatal B1 cells based on increased rates of self‐renewal, features of tonic BCR signaling, greater spontaneous IgM production, and shorter CDR3 junctions compared to other mature human B cell subtypes [245, 246], supporting the assertion that human B1 cells may be present in the CD27^+^ CD43^+^ population. In support of this, CXCR4 expression on circulating CD20^+^ CD27^+^ CD43^+^ B cells was associated with plasma levels of IgM to MDA‐LDL and inversely associated with human coronary artery plaque burden [23], suggesting that cells that produce atheroprotective IgM^OSE^ antibodies may reside within the CD20^+^ CD27^+^ CD43^+^ B cell population.

A more recent approach to identify the human cell type that produces IgM^OSE^ used an unbiased single‐cell mass cytometry approach [247]. Sort purified B cells from human subjects with high and low levels of IgM^MDA‐LDL^ were analyzed by mass cytometry (CyTOF). Eleven clusters of B cells were identified and tested for association with high levels of IgM^MDA‐LDL^. Only two B cell clusters characterized by CD27^+^ IgM^+^ were shown to be associated with high IgM^MDA‐LDL^. Within the CD27^+^ IgM^+^ population, the amount of CD24 on the surface was significantly associated with plasma IgM^MDA‐LDL^. Adoptive transfer of this human B cell subtype into NOD.scid gamma mice revealed that CD24^hi^ CD27^+^ IgM^+^ B cells trafficked to the spleen and produced IgM^OSE^ in vivo. Importantly, the phenotypic profile of this population aligned with that of circulating MZ B cells in humans (CD27^+^ IgM^+^ CD21^+^ CD23^−^ CD1c^+^), suggesting that human circulating MZ B cells are a source of protective IgM^OSE^ antibodies [247]. Moreover, the frequency of these CD24^hi^ circulating MZ B‐like cells was found to be inversely associated with CAD severity [247]. This insight not only identifies a specific human B cell subset as a source of IgM^OSE^, but also underscores the potential of human MZ B cells in modulating atherosclerosis. The presence of human MZ B cells has long been established; however, their role in regulating atherosclerosis development and progression is only now emerging. Important work by Tull et al. [248] established that human MZ B cells differentiate from early IgM^high^ transitional 2 B cells (T2) progenitors and can be identified by CD27^+^ IgM^+^ IgD^+^ surface marker expression. Unlike the MZ B cells in murine systems, human MZ B cells are not restricted to the spleen; rather, they circulate to other antibody‐producing niches such as intestinal Peyer's patches, extrafollicular lymphoid tissues, and tonsillar crypts [249, 250, 251, 252]. There, they display a partially activated phenotype that suggests an ability to rapidly produce antibodies upon antigenic stimulation [234, 251, 252, 253]. Human MZ B cells are phenotypically similar to unswitched memory B cells, expressing CD27 (a memory cell marker) and capable of class‐switching and producing IgG following immunization. This phenotypic similarity implicates an atheroprotective role for human MZ B cells, as Meeuwsen et al. [254] found that higher numbers of unswitched memory B cells, likely including human MZ B cells, were associated with better outcomes for patients with advanced atherosclerotic disease. Additionally, compelling work by Harrison et al. [242] found a significant correlation between human circulating MZ B cells (CD27^+^IgD^+^ unswitched memory B cells) and IgM^OSE^, including CuOx‐LDL and MDA‐LDL. These studies provide evidence for a protective role for human MZ B cells in atherosclerotic disease through the production of atheroprotective antibodies. However, as with murine MZ B cells, more research is required to fully characterize their mechanism of antibody production and their potential antibody‐independent roles in CVD.

#### 4.1.5 Metabolic Regulation of B1 and Marginal Zone B Cells

**FIGURE 3:**
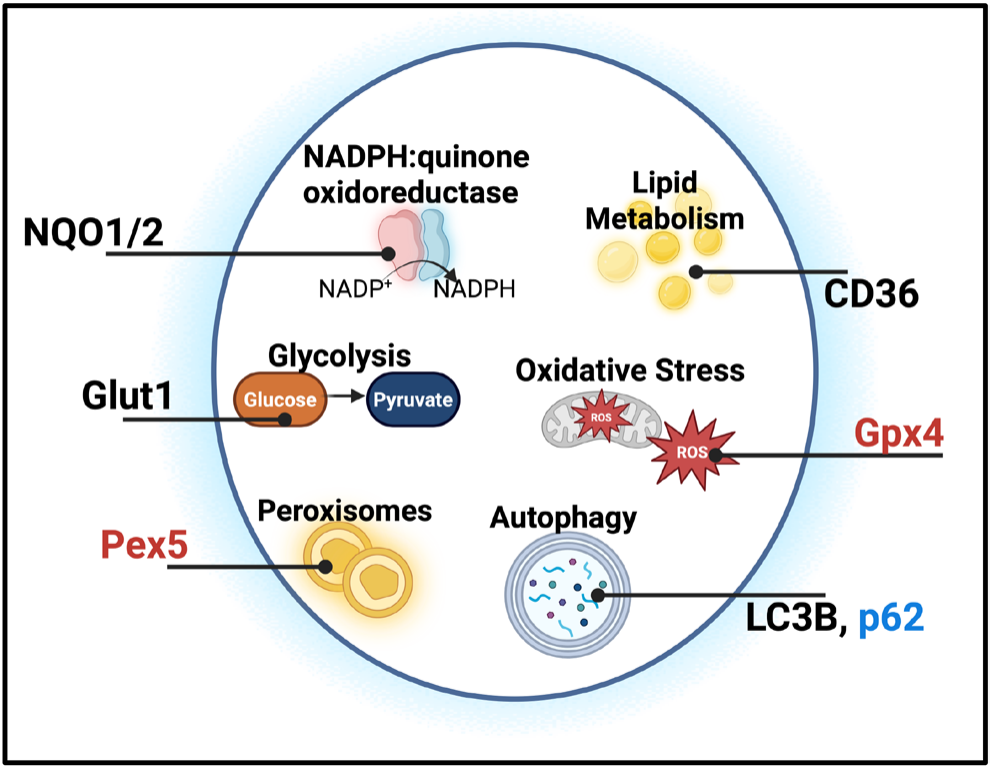
Schematic of metabolic regulation of B1 and marginal zone B cells. Metabolic homeostasis is key for B cell survival and function. B1 and marginal zone (MZ) B cell functions have greater dependence on endogenous antioxidant systems than follicular B cells. Processes that dysregulate B1 (blue) and both B1 and MZ B cells (red) redox homeostasis affects their ability to exact their effector function, including disruption of NADPH: quinone oxidoreductase 1/2 (NQO1 and NQO2), Gpx4 and Pex5, which protect B1 cells from cell death due to excess reactive oxygen species. Moreover, B1 and MZ B cells have higher rates of lipid metabolism and fatty acid uptake with a reliance on autophagy, shown to be partially regulated by CD36, a scavenger receptor, in MZ B cells and p62 and LC3B in B1 cells. Dysregulation of the pathways has significant impacts on self‐renewal, total and antigen‐specific IgM responses, and antigenic signaling strength through both the B cell receptor and toll‐like receptors.

Unlike other B cell subsets, B1 and MZ B cells have unique metabolic plasticity with a higher dependence on the regulation of autophagy, lipid metabolism, and endogenous antioxidant systems [246]. Reactive oxygen species (ROS) are generated by a variety of cellular metabolic processes, including mitochondrial respiration and antigenic engagement with TLRs and BCRs [255, 256]. These controlled fluxes of ROS have been shown to enhance activation and humoral immune responses in B cells [255, 256, 257, 258], with B1 and MZ B cells more susceptible to dysregulation of redox homeostasis. B1 cells have higher glucose uptake with elevated expression of glucose transporter type 1 (Glut1), rates of glycolysis and oxidative phosphorylation, and higher sensitivity to glycolytic inhibition than FO B cells [246] (Figure 3). Additionally, B1 and MZ B cells, but not FO B cells, have a particular reliance on glutathione peroxidases and peroxisomes, specifically Gpx4 and Pex5, which protect B1 cells from cell death due to excess ROS generated by lipid peroxidation (Figure 3) [259, 260]. In both a B cell‐specific Gpx4 knock‐out model (*Gpx4BKO*) and a Pex5 global knock‐out model (*Pex5KO*), B1 cells and MZ B cells specifically reduced their population size and proliferative capacity [259, 260]. Moreover, loss of Pex5 or Gpx4 resulted in reduced PC‐specific IgM production [259, 260], suggesting that T15 idiotype‐specific antibody production may be regulated by metabolism and redox homeostasis. Work by Ogura et al. [261] helps bolster this claim, demonstrating that excess mitochondrial ROS accumulation suppresses murine antibody responses. Moreover, NADPH:quinone oxidoreductase 1/2 (NQO1 and NQO2), two important antioxidant enzymes, have been shown to regulate antibody responses following TI antigen immunization, implicating both B1 and MZ B cells [262] (Figure 3). This suggests that disruption of redox homeostasis in B1 and MZ B cells may disrupt their IgM^OSE^ antibody responses and therefore influence atherogenesis. This novel area of research may have important clinical implications in characterizing risk for atherosclerosis based on factors that may disrupt redox homeostasis in innate B cells.

Beyond redox homeostasis, another function intertwined with metabolism that has been shown to regulate B cell production of IgM is autophagy. B1 cells are particularly reliant on self‐renewal to maintain consistent populations within their homeostatic niches, including the lipid‐rich peritoneal cavity [246]. Interesting work by Clarke et al. [246] showed that B1a cells have adopted a unique metabolic program, highly dependent on fatty acid uptake and high rates of glycolysis to support their self‐renewal and antibody production (Figure 3). Additionally, studies have shown that in B1b cells, p62, and autophagy protein, is a key regulator of IgM^OSE^ production and cellular proliferation [263]. Building upon this finding, work from He et al. [264] demonstrated that autophagy, specifically in MZ B cells, regulated their response to both TD and TI antigens, in part through CD36, a scavenger receptor, and LC3B, an autophagosome membrane protein (Figure 3). Finally, increased autophagy influences TLR9 activation of B cells and selective expansion of B1 cells [265]. While these studies do not specifically interrogate the role of autophagy in regulating IgM^OSE^ antibody responses, they point to a potential innate B cell‐specific program that may facilitate these atheroprotective antibody responses and thereby influence disease progression. Innate B cells clearly have distinct metabolic requirements and plasticity compared with FO and GC B cells, highlighting the potential to manipulate their metabolism to generate a more potent atheroprotective immune response.

### 4.2 Follicular and Germinal Center B Cells

#### 4.2.1 Murine Follicular and Germinal Center B Cells

FO B cells (CD19^+^ B220^+^ CD43^−^ IgM^low^ IgD^hi^ CD23^+^ CD21^int^ CD1d^−^ [266]) primarily reside in the follicles of SLOs, such as the spleen and lymph nodes, yet they retain the ability to circulate and migrate to distant sites such as the BM [267]. After activation by cognate CD4^+^ T cells, FO B cells can differentiate into GC B cells and participate in GC responses, or they develop via the EF pathway [268]. Both routes can lead to their differentiation into plasma cells producing class‐switched IgG, IgA, and IgE antibodies, which is considered the main mechanism by which FO B cells contribute to atherosclerosis development [65]. Previously, FO B cell reduction through B‐ and T‐lymphocyte attenuator (BTLA) stimulation significantly attenuated initial lesion development and increased plaque stability in advanced lesions of *Ldlr* ^*−/−*^ mice, indicating a pro‐atherogenic role for FO B cells [269]. This protection was likely not mediated by changes in humoral immunity, as neither total nor MDA‐LDL and ox‐LDL serum levels were changed, but increased apoptosis and reduced activation were observed in the FO B cells. To dissect the role of plasma cells derived from FO B cells in atherosclerosis, Tay et al. [65] created a mouse model lacking these cells through deletion of *Prdm1* (*CD23* ^*cre*^ *Prdm1* ^*flox*^ BM cells transplanted into *Ldlr* ^*−/−*^ mice). These mice exhibited greatly reduced plasma cell numbers and IgG and IgM levels, as well as attenuated atherosclerotic lesions, suggesting a pro‐atherogenic role for FO B cell‐derived plasma cells. Furthermore, they showed that the atherogenic function of FO B cells is highly dependent on the expression of co‐stimulatory molecule CD40 and MHC‐II, through which they interact with T(f)h cells in GC or EF reactions. In contrast, aggravated atherosclerosis development was observed after selective deletion of *Prdm1* in AID‐expressing cells (*Aicda* ^*cre*^ *Prdm1* ^*flox*^ BM cells transplanted into *Ldlr* ^*−/−*^ mice), which prevents the formation of high‐affinity class‐switched antibody‐producing plasma cells (mostly GC B cells), while keeping antibodies derived from B1 and nonclass‐switched B2 cells [270]. This suggests a potential atheroprotective function for class‐switched plasma cells. Consistently, IgGs have been implicated in plaque stabilization through inducing smooth muscle cell proliferation via FcγR signaling, suggesting that IgGs can exert protective effects in atherosclerosis [61]. Notably, in addition to IgGs, atheroprotective IgM levels were also reduced in the *Aicda* ^*cre*^ *Prdm1* ^*flox*^ *Ldlr* ^*−/−*^ chimeras; however, a considerable portion of IgM^+^ plasma cells remained, which were presumably derived from EF B cells [270]. On the other hand, AID deletion in *Apoe* ^*−/−*^ *Aid* ^*−/−*^ or selective loss of AID‐expressing B cells and resulting plasma cells in *Aicda* ^*Cre*^ *Pax5* ^*flox*^ *Apoe* ^*−/−*^ mice leads to decreased atherosclerosis [61, 103, 271]. Alongside this, total and autoantibody IgG titers were strongly reduced, while IgM levels were increased or not affected, respectively, implicating a proatherogenic role for IgG‐producing B cells. Although the reasons for the differing outcomes in these models remain unclear, they may reflect differences in the balance of antibody isotypes and antigen‐specificities.

The previous studies have mainly focused on the systemic contribution of FO or GC B cells or their derived plasma cells in atherosclerosis development, but less is known about how the local microenvironment within the lesion influences GC responses. In advanced atherosclerotic lesions in aged (18 months) *Apoe* ^*−/−*^ mice, adventitial ATLOs can emerge and harbor FO and GC B cells as well as plasma cells, which can contribute to local immune responses [20]. Recently, Zhang et al. [117] performed single‐cell RNA and BCR sequencing on immune cells derived from ATLOs and renal lymph nodes (RLNs) from aged (18 months) *Apoe* ^*−/−*^ mice and generated recombinant antibodies from BCR sequences of GC B cells to assess their reactivity against atherosclerotic lesions. Their findings revealed that ATLO‐derived antibodies have significantly higher plaque reactivity than those from RLNs, indicating that ATLO GCs preferentially promote the expansion of self‐reactive B cells. Moreover, the adoptive transfer of a histone 2B‐specific recombinant IgG2a antibody significantly aggravated atherosclerosis, underscoring the pathogenic potential of locally generated antibodies.

#### 4.2.2 Human Follicular and Germinal Center B Cells

Research on human FO B cells (CD19^+^ CD20^+^ IgD^+^ CD27^+/−^ [266]) in atherosclerosis remains limited, with most studies instead focusing on class‐switched B cell populations. Human atherosclerotic lesions have been shown to harbor IgG and IgA class‐switched B cells, plasmablasts, and plasma cells. For instance, Hamze et al. [15] reported that the majority of resident B cells found in the adventitia of CEA samples are mature B2 cell‐derived CD20^−^ plasmablasts, frequently expressing IgA or IgG isotypes as determined with RT‐PCR. This is supported by spatial transcriptomic data on carotid and coronary arteries showing B cells and plasma cells in the plaque and adventitia expressing IgA or IgGs [133, 272]. Lai et al. [133] recently reported lymphoid cell aggregates containing B cells and plasma cells within atherosclerotic plaques, which they termed plaque tertiary lymphoid organs (PTLO). Bulk RNA‐seq indicated that PTLO signature genes were enriched for B cell activation and immunoglobulin production pathways, although these structures lacked canonical features of GCs, such as CD21^+^ FDCs. Notably, the presence of PTLO structures correlated with symptomatic plaques. While intriguing, these observations were derived from a limited number of plaque sections and patients, warranting further investigation in a larger cohort to validate these findings.

Next to resident B cells, circulating B cell subsets have been identified and associated with CVD risk. High levels of unswitched (CD27^+^ CD43^−^ IgD^+^) and switched (CD27^+^ CD43^−^ IgD^−^) memory B cells at baseline were associated with a lower risk of secondary cardiovascular events in patients undergoing CEA, suggesting a protective role for these cells [254]. Furthermore, levels of switched memory B cells positively correlated with IgG levels specific for oxLDL, indicating that these cells are also responsible for IgG production. In contrast to these findings, no differences in frequencies of circulating unswitched and switched memory B cells have been observed between CVD patients and age‐matched healthy controls [273]. Another study reported increased plasmablast levels in CVD patients compared to low CVD risk individuals; however, the antibody isotype produced by these cells was not determined [274].

#### 4.2.3 Follicular and Germinal Center B Cell Communication via Cytokines

The contribution of FO B cell‐derived cytokines to atherosclerosis development remains largely undetermined. Tay et al. [275] showed that approximately 20% of splenic and aortic B cells in high‐fat diet‐fed *Apoe* ^*−/−*^ mice produce TNFα, with production levels exceeding those observed in chow diet‐fed mice. To further assess the role of TNFα‐deficient B cells in plaque development, a chimeric BM transplantation model (80% μMT^−/−^/20% TNFα^−/−^) was created, in which reduced lesion size and necrotic core size were observed. This effect was linked to decreased TNFα‐induced macrophage activation and subsequent secondary TNFα production, combined with decreased apoptosis, which TNFα can promote by inducing proapoptotic Fas expression on aortic smooth muscle cells. Adding to this, adoptive transfer of TNFα‐deficient B2 cells did not aggravate lesion development in *Apoe* ^*−/−*^ μMT^−/−^ or *Apoe* ^*−/−*^ Rag2^−/−^ γc^−/−^ mice, while WT B2 cells increased plaque size, pointing to a TNFα‐dependent mechanism of B2 cell‐driven atherosclerosis [275]. Supporting translatability, TNFα‐producing B2 cells have also been identified in human CEA samples [15]. Furthermore, IL‐6, which can be produced by activated FO B cells, induces Tfh cell differentiation and subsequent IL‐21 secretion, thereby promoting GC B cell formation [276]. This pathway has been identified as a key mechanism through which B cells initiate GC reactions and drive autoimmunity in systemic lupus erythematosus (SLE) mice [277], but its relevance in atherosclerosis is still unclear.

#### 4.2.4 Follicular and Germinal Center B Cell Communication via Immune Checkpoints

The CD40‐CD40L interaction is one of the primary stimulatory pathways of FO B cells. It is required for the proliferation and differentiation of B cells, initially in EF responses and later in the GC reaction with Tfh cells, where subsequent class‐switching, SMH, and formation of LLPCs and memory B cells are induced [278]. Selective deletion of CD40 in B cells by using a chimeric BM transplantation model (80% μMT^−/−^/20% CD40^−/−^) was shown to reduce GC B cells, Tfh cells, and IgG levels, but not IgM levels [65]. These mice exhibited decreased lesion size, suggesting a proatherogenic role for CD40^+^ B cells. In line with this, adoptively transferred CD40‐deficient B2 cells failed to increase lesion size in B cell‐deficient *Apoe* ^*−/−*^ mice, unlike wild‐type B2 cells, which increased plaque size by 109% [183]. In humans, on the other hand, high frequencies of CD40^+^ CD19^+^ B cells in circulation were linked to a lower incidence of stroke in patients, indicating a protective effect [279]. Although it was not determined to which subset of B cells this CD40^+^ B cells originated, it is noteworthy that CD40 signaling has been suggested to play a role in Breg activity [30, 280]. Further studies of the role of CD40 in specific B cell subtypes are needed to reconcile the contrasting findings.

As a consequence of CD40 stimulation, inducible T cell co‐stimulator (ICOS) ligand (ICOSL) is upregulated on GC B cells, which increases engagement with ICOS‐expressing GC Tfh cells, thereby maintaining GC Tfh cell function [281]. This signaling pathway is also important for differentiation of CD4^+^ T cells into Tfh cells, but this process relies on DCs [282]. In the context of atherosclerosis, studies determining this checkpoint have not been exclusive to B–T cell interactions, making its specific contribution difficult to assess. Nevertheless, scRNAseq on human atherosclerotic plaques revealed ICOSL‐expressing B cells and ICOS‐expressing CD4^+^ T cells [283], supporting a potential role for ICOS‐ICOSL interactions in local adaptive responses. In experimental models, antibody‐mediated targeting of ICOSL has yielded conflicting results. While Gaddis et al. [284] showed a reduction in atherosclerosis in *Ldlr* ^*−/−*^ mice due to decreased Tfh and GC B cells, Nus et al. [240] and Clement et al. [285] showed no effects on plaques in *Ldlr* ^*−/−*^ and *Apoe* ^*−/−*^ mice. Notably, the role of B cell‐specific ICOSL has been explored in autoimmune context in rheumatoid arthritis (RA) [286, 287]. Chimeric RA mice with ICOSL‐deficient B cells showed reduced percentages of Tfh cells and suppressed the production of T effector cell cytokines IFNγ and IL‐17. Furthermore, circulating ICOSL^+^ B cell levels as well as ICOS^+^ CD4^+^ T cell levels are increased in patients with RA compared to healthy controls and their levels positively correlate with clinicopathological characteristics [286].

Besides ICOSL, CD80 and CD86 molecules are also upregulated on FO B cells by CD40 stimulation [288], which can interact with CD28 or CTLA‐4 on T cells, resulting in stimulatory or inhibitory signaling, respectively. Although (FO) B cell specific studies on CD80/86 are limited, deficiency of CD80 and CD86 in *Apoe* ^*−/−*^ mice revealed reduced T‐effector cell responses and plaque size, suggesting an overall proatherogenic role for these checkpoints [289]. In line with this, high frequencies of CD86^+^ CD19^+^ B cells were associated with increased risk of stroke in CVD patients [279].

BTLA is another checkpoint that is highly and predominantly expressed on FO B cells and can exert its inhibiting function by interacting with herpesvirus entry mediator on, for example, T cells or DCs [269, 290]. As also discussed above (Section 4.2.1), Douna et al. [269] showed that activation of this pathway in *Ldlr* ^*−/−*^ mice reduced atherosclerosis, accompanied by expansion of Tregs and Th17 cells, indicating that BTLA treatment polarized CD4^+^ T cells toward an atheroprotective phenotype. Building on these preclinical findings, 90% of circulating B cells from CVD patients expressed BTLA, whereas this expression was markedly reduced in B cells present in matched CEA samples. Furthermore, a recent study using scRNAseq data to map the immune checkpoint landscape in human carotid and coronary atherosclerotic plaques predicted interactions between BTLA^+^ B cell and several T cell subsets [283]. This study also showed that a specific subcluster of TCL1A^+^ immature B cells exhibited increased CD200 expression compared to other immune cell subsets, while its receptor CD200R was mostly expressed on T cells and some natural killer (NK) cell and innate lymphoid cell subclusters [283]. In the circulation of CVD patients, CD200 expression was found to be the highest on B cells compared to T cells, NK cells, and monocytes, while CD200R was predominantly expressed by classical and intermediate monocytes [291]. The role of this inhibitory immune checkpoint pathway in atherosclerosis appears to be atheroprotective, as CD200R expression on circulating classical monocytes of CVD patients was found to be inversely correlated with plaque burden and deficiency of CD200 in *Apoe* ^*−/−*^ exacerbated atherosclerosis development [291].

Thus, FO B cells likely influence atherosclerosis development through the expression of various immune checkpoints that regulate T cells and other immune subsets. Although a deeper understanding of the exact role of each checkpoint is still needed, modulating these pathways through immune checkpoint therapies could be a promising approach to alter atherosclerosis progression.

### 4.3 Age‐Associated B Cells

An emerging B cell subset implicated in ASCVD is the ABC. Although originally identified as a distinct population expanding with age in both mice and humans [292, 293], ABCs have also been shown to accumulate independently of age during viral infections and in autoimmune diseases such as SLE and RA, in which their frequency correlates with disease activity [294, 295, 296, 297, 298]. Despite their established role in aging and autoimmunity (reviewed in Refs [299, 300]), the contribution of ABCs to ASCVD is only beginning to be explored.

#### 4.3.1 Murine Age‐Associated B Cells

In mice, ABCs are considered a heterogeneous subset of antigen‐experienced B cells characterized by low expression of CD21 and CD23, along with diverse expression of the myeloid markers CD11b and CD11c and the Th1 transcription factor T‐bet [301, 302]. Furthermore, Zeb2 was recently identified as a crucial transcription factor for ABC formation, supporting an EF origin for these cells [303]. Within the atherosclerotic lesion, all essential cues for ABC differentiation and activation are present, including necrotic debris‐derived nuclear antigens that activate TLR7/9, Th1‐derived cytokines, and T cells providing additional co‐stimulation [293, 304]. Accordingly, the presence of CD11b/c^+^ ABCs has recently been discovered in atherosclerotic aortas of chow diet‐fed 22‐month‐old *Ldlr* ^*−/−*^ mice through scRNAseq and flow cytometry, while being largely absent in 5‐month‐old Western‐type diet‐fed *Ldlr* ^*−/−*^ mice [305]. These ABCs displayed a distinct transcriptional profile compared to other aortic B cells, including elevated expression of *Tbx21* (encoding T‐bet) and plasma cell differentiation‐associated gene *Zbtb20*, suggesting a transition toward a plasma cell phenotype. In line with these findings, CD11b^+^ and CD11c^+^ ABCs were shown to increase systemically across several organs with aging in PCSK9‐induced atherosclerotic C57BL/6 mice [306] and in *Apoe* ^*−/−*^ mice [93], respectively, coinciding with increased aortic plaque burden. Moreover, ABC numbers are elevated in atherosclerotic mouse models compared to age‐matched C57BL/6 controls, with higher abundance of aortic CD11b/c^+^ ABCs observed in *Ldlr* ^*−/−*^ [305], and increased frequency of class‐switched CD21^−^CD23^−^CD11c^+^T‐bet^+^ ABCs in the spleen of 6‐month‐old *Apoe* ^*−/−*^ mice [307]. Together, these studies indicate that an atherosclerotic environment can promote the expansion of ABCs.

To determine how ABCs contribute to atherosclerosis development, Knox et al. [307] created an *Apoe* ^*−/−*^ mouse model with a B cell‐specific T‐bet deletion, in which significantly reduced amounts of CD21‐CD23^−^CD11c^+^ ABCs were detected compared to Cre control mice at the age of 6 months after 10 weeks of high‐fat diet. As a consequence, aortic lesion area was significantly reduced, thereby indicating a pro‐atherogenic role for ABCs. This was likely mediated by the accompanied decrease in IgG2c antibodies, reflecting the requirement of T‐bet for IgG2c class‐switching, while IgG1 levels were increased. However, since T‐bet deletion also affects IgG2c production beyond ABCs, it remains unclear to what extent autoantibodies produced by ABCs drive disease progression. Moreover, at 6 months of age, the ABC population in the mice used in this study remains relatively limited compared to that of aged mice (~20 months), as also demonstrated by Smit et al. [305]. Therefore, it would be interesting to determine whether B cell‐specific T‐bet deletion confers even greater benefits at a more advanced age.

In addition to IgG2c, aortic ABCs display strong expression of genes encoding for IgG1 and IgG3 compared to other B cells, along with transcripts for IgM, IgA, and IgG2b [305, 308]. Yet, how this profile translates into antibody production and which antigens these antibodies recognize remain to be explored. Interestingly, females displayed a greater expansion of ABCs, accompanied by higher expression of immunoglobulin and plasma cell‐associated genes than males, suggesting a greater contribution for ABCs in atherosclerosis development in female mice [308].

Apart from their role in antibody production, ABCs shape adaptive immune responses by acting as potent APCs. Their high MHC‐II and CD86 expression enables enhanced T cell activation [292], as was also shown in vitro, where ABCs isolated from aged *Ldlr* ^*−/−*^ mice induced stronger antigen‐specific T cell activation and proliferation than FO B cells [305]. Furthermore, ABCs from atherosclerotic mice showed elevated expression of genes encoding pro‐inflammatory cytokines and chemokines such as TNFα, IL‐1β, and CCL2 compared to other B cells, as well as co‐stimulatory and ‐inhibitory immune checkpoints such as *Havcr1* (encoding Tim‐1), *Cd200r*, *Cd80*, *Tnfsf4* (encoding OX40L), and *Fas*, enabling crosstalk with surrounding immune cells [305].

#### 4.3.2 Human Age‐Associated B Cells

ABCs with similar phenotypic and functional characteristics as in mice have been described in the circulation of humans as CD11b/c^+^ [305], atypical (memory), CD21^low^, or double negative 2 (DN2, IgD^−^ CD27^−^ CXCR5^−^ CD21^−^ CD11c^+^ IgE^−^) B cells [300, 309], which likely represent overlapping populations. Circulating ABCs (CD21^−^ CD11c^+^) have been reported to independently predict the presence of atherosclerosis in patients with early RA and their frequency positively correlates with IL‐21, IFNγ, TNFα, and IL‐6 levels [310]. Consistent with these findings, Pattarabanjird et al. [93] identified CD27^−^IgD^−^CXCR5^−^ and CD27^low^ IgD^−^ CXCR5^−^ subsets of CD11c^+^ B cells in the circulation of CVD patients using CITE‐seq that were associated with severe atherosclerosis. The frequency of CD11c^+^ CD27^−^ IgD^−^ CXCR5^−^ B cells positively correlated with plasma levels of MDA‐mimotope‐specific IgG and pathway analysis of differentially expressed genes in patients with high CAD severity revealed enrichment of autophagy and IFNγ signaling pathways, thereby providing insights into potential mechanisms through which CD11c^+^ CD27^−^ IgD^−^ CXCR5^−^ B cells could exacerbate atherosclerosis. Further supporting their involvement in atherosclerosis, the research of Smit et al. [305] identified ABCs (CD11b/c^+^) in human carotid plaques using flow cytometry and showed that they are enriched in this compartment compared to the circulation. Recent scRNAseq and spatial transcriptomic analysis on CEA plaques identified the presence of FCRL3^+^ atypical memory‐like B cells within the lesions [133], characterized by enrichment of a previously described gene signature [311] of atypical memory B cells also observed in aortic ABCs [305, 308], including *ITGAX* (*CD11c*), *FCRL5*, *ZEB2*, and *FGR*. Additionally, intercellular communication analysis between these FCRL3^+^ B cells and Tfh cells using plaque scRNAseq data indicated high expression of several ligand‐receptor pathways involved in B cell recruitment and differentiation, including CD40‐CD40L and CXCL13–CXCR5. However, these observations were derived from only one patient and should therefore be interpreted with caution. While these findings provide potential new insights into the localization and communication of ABCs in the plaque, future studies are required to validate these findings on protein level and extend the analysis to other lesion‐associated compartments, such as ATLOs in the adventitia and FALCs in the PVAT, where B cells were shown to predominantly reside [15, 16, 19]. Moreover, further investigations should focus on elucidating the cellular origin, heterogeneity, intercellular communication, and autoantigen repertoire of ABCs in atherosclerosis to get a deeper understanding of their involvement in disease progression.

**FIGURE 4:**
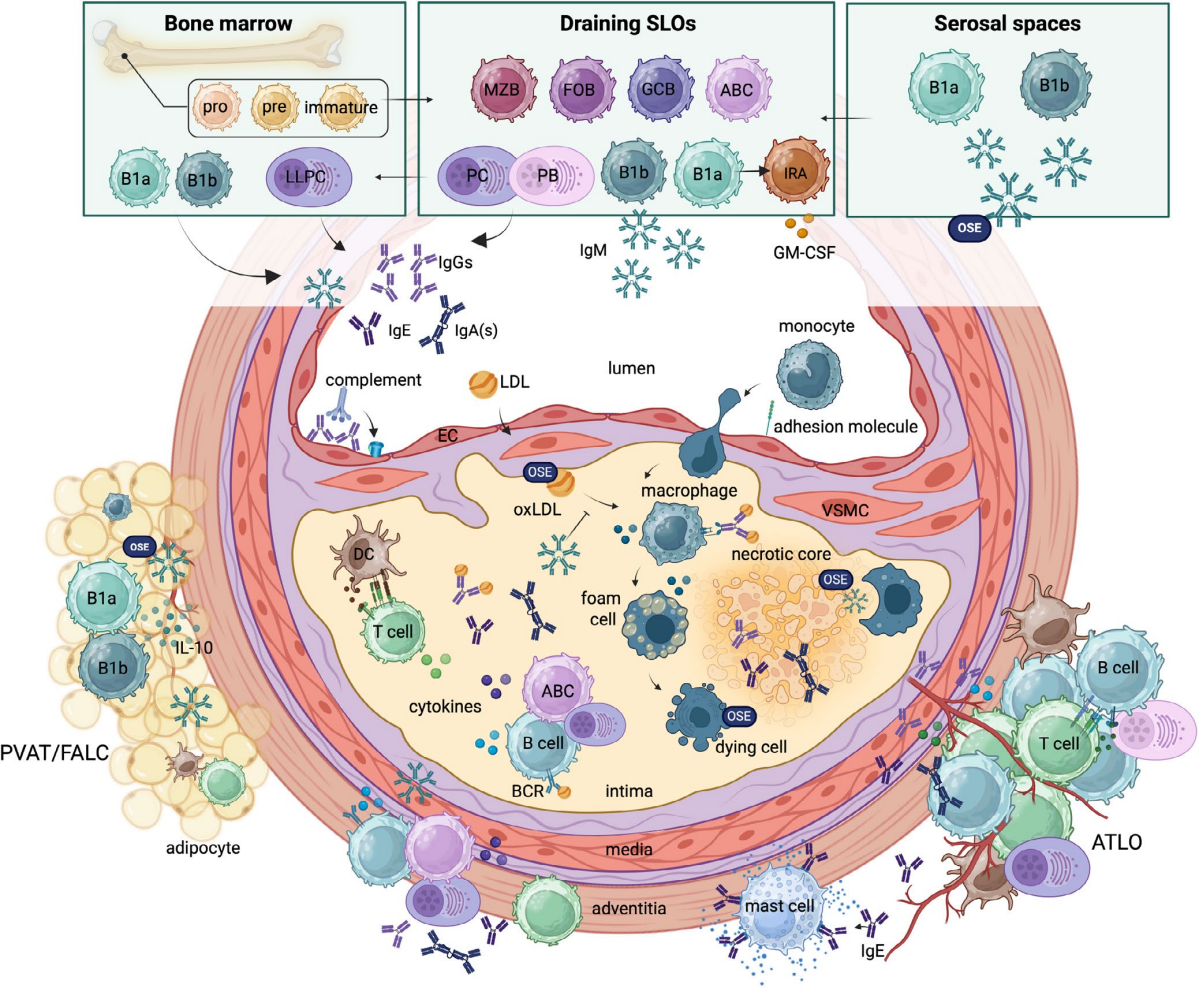
B cells in atherosclerotic cardiovascular disease. B1 cells, including B1a and B1b cells, predominantly develop in the fetal liver and reside in serosal spaces. Following antigenic stimulation, they can migrate to antibody producing niches, including the perivascular adipose tissue (PVAT), spleen and bone marrow. Within the PVAT, B1 cells can organize in fat‐associated lymphoid clusters (FALCs) together with other immune cells. In these niches, B1 cells secrete oxidation‐specific epitope (OSE)‐specific IgM and the anti‐inflammatory cytokine interleukin 10 (IL‐10). B1a cells can additionally give rise to pro‐atherogenic innate‐response activator (IRA) B cells, which produce granulocyte macrophage‐colony stimulating factor (GM‐CSF). B2 cells develop primarily in the bone marrow, where progenitor (pro) and precursor (pre) B cells differentiate into immature B cells. These cells further mature in secondary lymphoid organs (SLOs) and differentiate into marginal zone (MZ) or follicular (FO) B cells, the latter giving rise to germinal center (GC) B cells upon activation (Figure 1). With aging, age‐associated B cells (ABCs) emerge and, together with other B cells, can migrate to the plaque. Activated B cells differentiate into plasmablasts (PBs) and plasma cells (PCs) secreting immunoglobulin M (IgM), IgE, or subclasses of IgA or IgG. PCs can home to the bone marrow and become long‐lived plasma cells (LLPCs), secreting antibodies that reach the plaque via circulation. Atherosclerotic plaque development begins with deposition and retention of circulating lipids, particularly low‐density lipoprotein (LDL), within the arterial intima. There, LDL undergoes oxidation to form oxidized LDL (oxLDL), thereby generating OSEs that can be recognized as neo self‐antigens by immune cells. OxLDL can be taken up by infiltrating macrophages, which subsequently transform into foam cells producing pro‐inflammatory cytokines that recruit additional innate and adaptive immune cells, including T and B cells, to the plaque and surrounding media and adventitia. Progressive accumulation of dying foam cells and lipids gives rise to a necrotic core, providing a sustained source of OSEs fueling the ongoing inflammatory response. Antigens can be presented by antigen‐presenting cells such as dendritic cells (DCs) in the plaque, in draining SLOs, or in artery tertiary lymphoid organs (ATLOs) that form in the adventitia during advanced disease stages. In ATLOs, local B cell activation can give rise to PBs and PCs, resulting in the production of antibodies that can migrate to the plaque. Within the lesion, antibodies can form immune complexes with atherosclerotic antigens and exert diverse effector functions. IgM antibodies neutralize oxLDL, thereby preventing foam cell formation, can promote the clearance of dead cells through macrophages, and thereby limiting necrotic core formation. IgE exerts its pro‐atherogenic effects by activating mast cells through Fc epsilon receptor engagement. IgGs modulate immune cells by binding to their activating or inhibitory Fc gamma receptors, can neutralize antigens or facilitate their clearance via opsonization, with or without recruitment of the complement system, and can mediate cytotoxicity in a cell‐dependent or complement‐dependent manner. The contribution of IgA to atherosclerosis and the mechanisms involved remain unclear. VSMC, vascular smooth muscle cell; BCR, B cell receptor.

## 5 Therapies

The role of the humoral immune response and the different subtypes of B cells in atherosclerosis is an ever‐developing field. It is clear that all subtypes of B cells play unique roles in the pathogenesis of atherosclerosis, both atherogenic and atheroprotective (Figure 4), however, the full scope of their impact and regulation is not well understood. There have been major advances in the clinical treatment and prevention of ASCVD. Beyond risk factor management, modern clinical approaches seek to reduce cholesterol biosynthesis and LDL receptor activity, reduce lipoprotein lowering, and target inflammation (recently reviewed [312]). Despite these advancements, CVD is still the leading cause of death worldwide. As such, innovative approaches are needed. Targeting B cell biology may be a novel approach to improve therapeutic outcomes. This section of the review will give mention to a few approaches that are being considered and tested.

### 5.1 Vaccination Against Atherosclerosis

#### 5.1.1 IgM Targeted Vaccination

The notion that atheroprotective functions of innate B cells might be harnessed to protect against atherosclerotic disease has been of interest for some time. Perhaps the earliest example of this is through immunization of hyperlipidemic animal models with bacterial or modified lipid epitopes to increase the IgM^OSE^ response [79, 89, 313, 314]. Palinski et al. [313] foundationally showed that immunization of hyperlipidemic mice and rabbits resulted in reduced atherogenesis along with increased IgM^MDA‐LDL^. As many studies have shown that titers of IgM^MDA‐LDL^ are inversely associated with atherosclerosis, analyzed via coronary angiography, coronary artery calcium, and coronary events in humans [71, 75, 94, 315], MDA adducts may be important immunodominant epitopes for atheroprotective responses in humans. However, oxLDL epitopes, including MDA‐LDL, are not the only atherosclerotic antigens to be identified as immunogenic. In fact, Apolipoprotein B100 has been shown to be a potential therapeutic target and immunogen by a variety of studies [316]. Furthermore, many studies have identified that immunization with *S. pneumoniae* results in an expansion of anti‐PC IgM antibodies, suggesting that *S. pneumoniae* could be a potential immunogen by increasing the atheroprotective E06/T15 type antibody responses [81, 89, 317, 318]. Indeed, a study by Briles et al. [317] shows that immunization of toddlers with 9‐valent pneumococcal conjugate vaccine resulted in proportionally higher IgM to IgG titers, with perpetuation of active IgM for at least a year. These studies suggest that immunization of humans with atherosclerotic neoantigens may be a promising approach to prevent atherogenesis, although many questions remain.

#### 5.1.2 IgG Targeted Vaccination

While much ambiguity surrounds the role of IgG subclasses as pro‐ versus antiatherogenic, given the nature of the humoral immune response, IgG will be the most prominent Ig generated. Moreover, its longevity and high affinity offer the advantage of a more efficacious therapeutic response. One approach to neutralize pro‐atherogenic antigens and limit the stimulation of pro‐inflammatory pathways is through passive immunization with IgG. Preclinical studies of passive vaccination have shown contradicting roles of anti‐oxLDL IgG antibodies [65, 96]. The GLACIER trial was one of the first studies in humans to test the use of injectable immunoglobulins. This randomized, double‐blind phase II study tested the efficacy of anti‐oxLDL‐C monoclonal antibody in patients with stable carotid plaque or aortic plaque lesions [319]. Specifically, this study found that passive immunization with MLL1278A, an anti‐oxLDL antibody, added to lipid lowering therapies did not reduce cardiovascular events nor arterial inflammation, assessed via FDG‐PET imaging. This study was limited by its small sample size and limited longitudinal study period; however, the notion of passive immunization has not been forgone. Indeed, it was recently shown by Lorenzo et al. [97] that in mice, IgG2b antibodies specific for the self‐antigen ALDH4A1 can reduce circulating free cholesterol and LDL and thereby inhibit plaque progression. Anti‐PC IgG antibodies have been suggested to be efficacious in preclinical models in blocking macrophage engulfment of ox‐LDL‐C [146]. A current phase IIa, placebo‐controlled, double‐blind study tested the efficacy of a human IgG1 anti‐PC antibody (ATH3G10) in high‐risk patients with ST‐segment elevation myocardial infarction (STEMI); however, results are still pending. A recent promising placebo‐controlled phase 1a trial tested human IgG1 anti‐MDA‐ApoB100 (orticumab) in psoriasis patients and found that orticumab reduced coronary inflammation [320]. This study emphasizes the promiscuity of the anti‐ versus pro‐atherogenic role of IgG1 as well as providing potential efficacy of passive immunization strategies against atherosclerosis.

In addition to passive immunization, active immunization is a promising vaccination strategy; however, it presents many challenges including selecting which of the many atherosclerotic antigens to target, HLA restrictions, and balancing stimulatory versus tolerogenic responses. Stimulatory vaccines have sought to enhance protective immunity including Ig responses, Th2 polarization, or cytotoxic responses against pro‐inflammatory immune cells. Recently, a study showed that immunization with a nanovaccine with p210 and antigen epitope in human apolipoprotein B100 in murine models induces both anti‐p210 IgM and IgG antibodies to suppress atherosclerosis [321]. Additionally, in *Ldlr* ^*−/−*^ mice, immunization of MDA‐LDL in complete Freund's adjuvant activated a strong GC, plasma cell, and switched anti‐MDA‐LDL antibody response, eliciting atheroprotection that is lost in mice deficient in GC‐derived plasma cells [270]. Nonetheless, preclinical and early clinical studies collectively demonstrate that immune reprogramming is feasible and can modulate atherosclerosis, providing a foundation for next‐generation immunotherapies.

### 5.2 B Cell Depletion Therapy

B cell targeting therapy is in use to treat a wide variety of diseases such as SLE, RA, multiple sclerosis, B cell leukemias, and multiple myeloma. Studies in preclinical models [184, 322, 323, 324] suggest that biological therapeutics that deplete B cells and plasma cells including rituximab‐CD20, ofatumumab‐CD20, ocrelizumab‐CD20, blinatomumab‐CD19, inebilizumab‐CD19, inotuzumab‐CD22, ozogamicin‐CD22, elotuzumab‐SLAMF7, daratumumab‐CD38, GSK2857916‐BCMA, and AMG420/BI836909‐BCMA [325, 326], may be impactful and efficacious in the context of human atherosclerosis. However, it is unclear how each of these B cell depletion therapies might impact the different B cell subtypes, more specifically, innate‐like B cells. Interestingly, in murine studies by Ait‐Oufella et al. [184] and Kyaw et al. [322, 323], treatment of mice with CD20‐specific monoclonal depletion antibody resulted in reduced diet‐induced atherosclerosis and a preferential reduction in B2 cells, while only affecting 30%–40% of B1 cells. Sage et al. [327] impaired B cell survival by blocking the B cell activating factor (BAFF) and one of its receptors (BAFFR) and showed a significant reduction in B2 cells, but not in B1a cells and IgM antibody titers in hyperlipidemic mice. There are many BAFF/BAFFR and APRIL (A Proliferating‐Inducing Ligand) targeting therapeutics currently utilized in clinic to treat a variety of autoimmune conditions [328, 329, 330, 331, 332, 333, 334]. However, to date, no B cell subtype‐specific effects or impact on CVD have been reported. Studying patients receiving these therapies for these endpoints may be informative.

## 6 Concluding Perspectives

B cells have been implicated in atherosclerosis for over a century, yet only recently has the field begun to appreciate the full complexity of their contributions to atherogenesis. As this review highlights, the humoral immune response is far from simple; rather, it is composed of diverse B cell subsets with distinct, and often opposing, roles in atherogenesis (Figure 4). Defining the phenotypes, functions, and mechanisms of both pro‐ and antiatherogenic B cell populations in mice and humans remains essential for resolving long‐standing uncertainties in the field and identifying novel avenues for therapeutic intervention.

Particularly noteworthy are emerging insights into ABCs and the innate B cell compartment. B1 cells and MZ B cells support atheroprotection through the production of IgM antibodies that recognize atherosclerotic antigens including OSEs and dampen inflammation. Understanding how pathways such as redox homeostasis governs their function may facilitate opportunities to augment these endogenous defenses therapeutically. In contrast, ABCs, whose expansion is linked to aging, the strongest risk factor for ASCVD, have a multifaceted influence on atherogenesis through not only antibody production but also cytokine release and crosstalk with other immune cells. Their integration into broader immunologic networks positions them as compelling targets for intervention.

Despite substantial advances, the expanding knowledge of B cell biology in atherosclerosis raises as many questions as it answers. The field now faces the challenge of translating mechanistic insights into strategies that maintain immunological balance by selectively enhancing protective B cell responses while restraining pathogenic ones. Continued efforts to elucidate human B cell heterogeneity, define the antigens that shape these responses, and map their interactions within the systemic and atherosclerotic microenvironment will be critical. Ultimately, a deeper understanding of B cell immunity may not only clarify fundamental aspects of atherogenesis but also guide the development of more precise immunomodulatory therapies for CVD.

## Funding

The authors' research work covered in this review was supported by the Foundation Leducq Network of Excellence grant on Immune Checkpoints in Atherosclerosis (CHECKPOINT ATHERO, 22CVD02), the project “B‐specific” funded by the European Union under Grant Agreement No. 101115159, the project MARGINALIZE‐MI with file number era4healthcvd‐105 of the research program ERA4Health CARDINOVV which is (partly) financed by the Dutch Research Council (NWO), P01 HL136275 (CAM), R01 HL176892 (CAM), and Foundation Leducq grant number TNE‐20CVD03 (CAM).

## Conflicts of Interest

The authors declare no conflicts of interest.

## Data Availability

Data sharing is not applicable to this article as no datasets were generated or analyzed during this study.
